# Acceptance of clinical artificial intelligence among physicians and medical students: A systematic review with cross-sectional survey

**DOI:** 10.3389/fmed.2022.990604

**Published:** 2022-08-31

**Authors:** Mingyang Chen, Bo Zhang, Ziting Cai, Samuel Seery, Maria J. Gonzalez, Nasra M. Ali, Ran Ren, Youlin Qiao, Peng Xue, Yu Jiang

**Affiliations:** ^1^School of Population Medicine and Public Health, Chinese Academy of Medical Sciences and Peking Union Medical College, Beijing, China; ^2^Faculty of Health and Medicine, Division of Health Research, Lancaster University, Lancaster, United Kingdom; ^3^School of Public Health, Dalian Medical University, Dalian, China; ^4^The First Affiliated Hospital, Dalian Medical University, Dalian, China; ^5^Global Health Research Center, Dalian Medical University, Dalian, China

**Keywords:** artificial intelligence (AI), acceptance, physicians, medical students, attitude

## Abstract

**Background:**

Artificial intelligence (AI) needs to be accepted and understood by physicians and medical students, but few have systematically assessed their attitudes. We investigated clinical AI acceptance among physicians and medical students around the world to provide implementation guidance.

**Materials and methods:**

We conducted a two-stage study, involving a foundational systematic review of physician and medical student acceptance of clinical AI. This enabled us to design a suitable web-based questionnaire which was then distributed among practitioners and trainees around the world.

**Results:**

Sixty studies were included in this systematic review, and 758 respondents from 39 countries completed the online questionnaire. Five (62.50%) of eight studies reported 65% or higher awareness regarding the application of clinical AI. Although, only 10–30% had actually used AI and 26 (74.28%) of 35 studies suggested there was a lack of AI knowledge. Our questionnaire uncovered 38% awareness rate and 20% utility rate of clinical AI, although 53% lacked basic knowledge of clinical AI. Forty-five studies mentioned attitudes toward clinical AI, and over 60% from 38 (84.44%) studies were positive about AI, although they were also concerned about the potential for unpredictable, incorrect results. Seventy-seven percent were optimistic about the prospect of clinical AI. The support rate for the statement that AI could replace physicians ranged from 6 to 78% across 40 studies which mentioned this topic. Five studies recommended that efforts should be made to increase collaboration. Our questionnaire showed 68% disagreed that AI would become a surrogate physician, but believed it should assist in clinical decision-making. Participants with different identities, experience and from different countries hold similar but subtly different attitudes.

**Conclusion:**

Most physicians and medical students appear aware of the increasing application of clinical AI, but lack practical experience and related knowledge. Overall, participants have positive but reserved attitudes about AI. In spite of the mixed opinions around clinical AI becoming a surrogate physician, there was a consensus that collaborations between the two should be strengthened. Further education should be conducted to alleviate anxieties associated with change and adopting new technologies.

## Background

Artificial intelligence (AI) refers to machine-based systems which simulate problem-solving and decision-making processes involved in human thought. The success of Google’s AlphaGo program in 2016 propelled Deep Learning (DL) led AI into a new era, and stimulated interest in the development and implementation of AI systems in many fields, including healthcare. Between 1997 and 2015, fewer than 30 AI-enabled medical devices were approved by the U.S. Food and Drug Administration (FDA), however this number rose to more than 350 by mid-2021 (1). Also, there is an increasing number of studies which have found that DL algorithms are at least equivalent to clinicians in terms of diagnostic performance (2–4). This means that DL-enabled AI has the potential to provide a number of advantages in clinical care. For example, DL-enabled AI could be used to address current dilemmas such as the workforce shortage and could ensure there is consistency by reducing variability in medical practice and by standardizing the quality of care (5). Some have suggested that the increasing use of AI will fundamentally change the nature of healthcare provision and clinical practice (6–8). However, this gradual transition could also cause concerns within the medical profession because adopting new technologies requires changes to medical practice.

At present, the relatively limited use of clinical AI partly reflects a reluctance to change as well as potential misperceptions and negative attitudes held by physicians (9, 10). Of course, physicians are likely to be the “earliest” adopters and inevitably become direct AI operators. Therefore, physicians play a pivotal role in the acceptance and implementation of clinical AI, and so their views need to be explored and understood. AI-driven changes will also inevitably affect medical students, the future generations of doctors. Therefore, research should be designed to understand their sentiments in order to develop effective education and health policies. There is a growing evidence-base around the attitudes of physicians and medical students toward AI. However, there are distinctions between countries and cultures and the majority of this research has been conducted in developed, western countries (11, 12). While there has also been a couple of systematic reviews on this topic (9, 13), we can still say that this provides only a narrow understanding. There is a need to understand the views of medical students and physicians in developing countries in Asia and Africa. Therefore, we conducted a two-stage study, involving a foundational systematic review which enabled us to design a suitable questionnaire that was then distributed among physicians and medical students around worldwide. This approach was implemented to obtain more comprehensive data and to discuss contrasting ideas, in order to gain insights to improve the uptake and use of clinical AI.

## Materials and methods

We initially conducted a systematic review to understand what is already known about physicians’ and medical students’ perspectives on clinical AI. The initial systematic review followed rigorous procedures set out in the Preferred Reporting Items for PRISMA (Preferred reporting items for systematic reviews and meta-analysis) statement (14). The main themes, identified through the systematic review, were used to develop a questionnaire, which was then distributed through a network of associates.

STROBE checklist was provided for this cross-sectional study (15). Participation in the questionnaire was voluntary and informed consent was obtained before completing the questionnaire. The research ethics committee of Chinese Academy of Medical Sciences and Peking Union Medical College approved this study (IEC-2022-022).

### Systematic review

Clinical AI, during the systematic review stage, was defined as “AI designed to automate intelligent behaviors in clinical settings for the purpose of supporting physician-mediated care-related tasks”. These clinical AI technologies excluded consumer utilized products such as wearable devices. PUBMED, EMBASE, IEEE Xplore and Web of Science were systematically searched for published research. Any original study appraising physician or medical student acceptance of clinical AI, published in English from January 1st 2017 to March 6th 2022, was initially included. Conference abstracts and comments presenting conclusions without numerical data were excluded. Search strategies are listed in the Supplementary Material 1.

Bibliographic data obtained were loaded into Endnote (version 20) and duplicates were removed. Authors BZ and ZC independently reviewed titles and abstracts to identify pertinent research which met the established inclusion criteria. Full-text assessment was conducted for inclusion. BZ and ZC independently extracted data from each eligible study using a pre-designed template. Inconsistencies were resolved through discussion with MC.

### Questionnaire survey

A web-based questionnaire was generated based on the findings of the systematic review under the guidance of two experts in clinical AI. The draft questionnaire was then pre-tested across a sample of 110 students, and two participants were interviewed about their understanding of each question and about any difficulties met while completing the survey. The questionnaire was adjusted according to feedback from the pilot study (Supplementary Material 2).

The questionnaire was constructed around three main elements. The first section focused on respondent characteristics and practical experiences of clinical AI. The second included 13 statements to assess respondent’s views of clinical AI. These included aspects such as awareness and knowledge, acceptability, as well as AI as surrogate physicians. Respondents were asked to indicate their level of agreement with statements using a five-point Likert scale. In this instance, one was understood as strong disagreement while five was considered to be strong agreement with the statement. In the third section, respondents were asked to suggest factors which they feel are associated with intentionality, as well as around the perceived relationship between physicians and clinical AI. Section three was also designed to gain insights into the perceived challenges involved in the development and implementation of clinical AI. The online questionnaire was distributed among physicians and medical students through our professional network in March 2022.

### Statistical analysis

Continuous variables are presented as means with corresponding standard deviations. Categorical variables are described using frequencies and percentages. Differences between physicians and medical students in clinical AI practice was compared using a standard Chi-square test. Comparisons of the response distribution on 13 statements across subgroups were performed by Mann–Whitney *U* test.

For descriptive statistics categories “strongly disagree” and “disagree” were summarized as disagreement while “agree” and “strongly agree” were summarized as agreement. Correlations between demographics and a willingness to adopt clinical AI were assessed using multivariable logistic regression, in physicians and medical students separately. Under statistical analysis, the “willingness to use clinical AI” was dichotomized according to having responded “strongly agree or agree” as opposed to “neutral or disagree or strongly disagree” for statement “I am willing to use clinical AI if needed”. All statistical analyses were performed using R (version 4.1.0). A *p* value <0.05 was established as the threshold for statistical significance.

## Results

### Description of included studies and respondent characteristics

**FIGURE 1:**
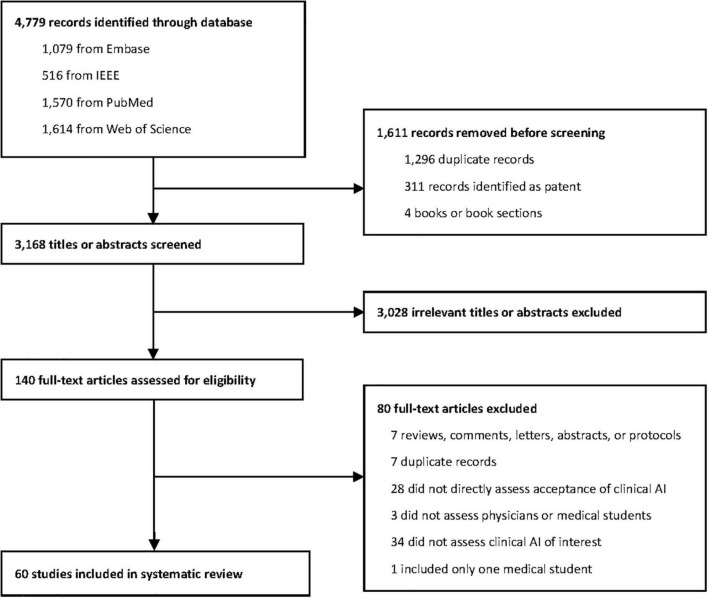
PRISMA (Preferred reporting items for systematic reviews and meta-analysis) systematic review flow diagram. Displayed is the PRISMA flow of each article selection process.

**FIGURE 2:**
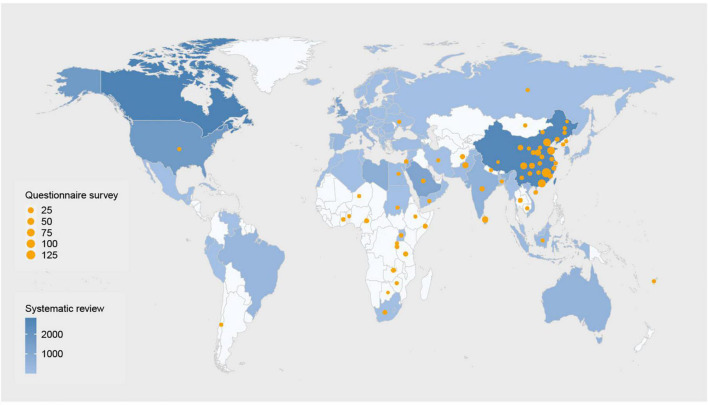
Geographic distribution of participants in the systematic review and the survey. The blue indicates the number of participants of studies included in the systematic review. The darker the color, the more participants. The orange dots indicate the number of participants in our questionnaire survey. The larger the dots, the more participants. Studies without providing specific locations are not shown in the figure. Please see Table 1 for detailed number and locations of participants.

Figure 1 provides the Systematic reviews and Meta-Analyses (PRISMA) flow diagram of this systematic review. Characteristics and main findings of the included studies have been summarized in Table 1 and Supplementary Table 1. Of the 60 included studies, there were 47 (78%) quantitative studies, 7 (12%) qualitative studies, and 6 (10%) mixed methods studies. All studies were published between 2019 and 2022. In the study population, 41 (68%) studies recruited physicians, 13 (22%) studies surveyed medical students, and 6 (10%) studies included both physicians and medical students. Regarding the type of AI being studied, 20 (33%) studies assessed AI in radiology, 13 (22%) assessed AI that was broadly defined, 9 (15%) assessed AI-based decision support system in clinic, 5 (8%) for AI in dermatology, 3 (5%) for AI in gastroenterology, 2 (3%) for AI in ophthalmology, and 2 (3%) for AI in psychiatry, etc. 35 (58%) studies were conducted in high-income countries, 6 (10%) were conducted in upper-middle income countries, 4 (7%) in lower-middle income countries, and 13 (21%) were conducted worldwide or regionally. The geographical distribution of included studies is presented in Figure 2.

**TABLE 1:** Characteristics of studies included in the systematic review.

References	Study design	Study population and location	Number of participants	Participant characteristics	Artificial intelligence (AI) studied
Shelmerdine et al. (48)	Quantitative	Memberships of ESPR, SPR, ANZSPR, BMUS and SoR, mainly in Europe	240	59% aged 30–49 years; 52.1% female; 66.3% radiologists, 31.3% allied health care professionals, and 2.5% non-medical background	AI in pediatric radiology
Buck et al. (28)	Qualitative	General practitioners, in Germany	18	Mean age 47.33 years (range 34–70, SD 8.31); 50% female; all with at least 1 year of work experience in GP care; 39% in rural areas	AI-based systems in diagnosis
Abuzaid et al. (16)	Quantitative	Radiology professionals (radiologists and radiographers) working in radiology departments, in United Arab Emirates	153	Mean age of radiographers and radiologists 35 and 43 years, respectively; 35.3% female; 77.8% radiographers and 22.2% radiologists; 55.9% master’s degree and 44.1% Ph.D. qualified radiologists, 79.0% bachelor’s degree and 11.8% masters degree qualified radiographers	AI in radiology
Khafaji et al. (29)	Quantitative	Radiology residents enrolled in the diagnostic radiology training program, in Saudi Arabia	154	44.8% female; 48.7% from the central region; 25.9% in the first year of training, 33.8% in the third year	AI in radiology
Lim et al. (69)	Quantitative	Non-radiologist clinicians at a tertiary referral hospital in Melbourne, VIC, Australia	88	Median age (IQR 31–40); 22.7% female; 77.3% consultants, 22.7% doctors-in-training	AI in diagnostic medical imaging reports
Kansal et al. (30)	Quantitative	Doctors and medical students in Punjab state, northern India	367	40.6% female of medical students, 41.9% female of doctors; 34.9% third-year medical students	AI in medicine, broadly defined
Eiroa et al. (31)	Quantitative	Radiologists (residents and attending physicians), in Spain	223	76.7% attending physicians, 23.3% residents; 50.9% of attending physicians in the public setting; 63.5% of residents with desire to work in the public setting	AI in radiology, imaging informatics
Reeder and Lee (27)	Quantitative	Students across 32 allopathic medical schools, in the USA	463	43.2% female; 64.6% in the first and second year; 20.5% ranking radiology as fourth or lower choice; 22.5% and 29.2% interested in diagnostic and interventional radiology, respectively	AI in medicine, broadly defined
Teng et al. (32)	Mixed methods	Health care students across 10 different health professions from 18 universities enrolled in an entry-to-practice health care program, in Canada	2167	56.16% aged 21–25 years; 62.53% female; 31.52% from medical doctorate program, 23.72% from nursing program; 53.53% bachelor’s degree	AI in medicine, broadly defined
Pangti et al. (49)	Quantitative	Dermatologists and dermatology trainees, in India	166	Mean age 36.45 years (range 23–69, SD 13); 40.4% female; mean duration of experience 7.80 years (SD 10.92); 28.3% in government hospitals, 29.5% in private hospitals or clinics	AI in dermatology
Leenhardt et al. (24)	Quantitative	Gastroenterologists, in 20 European countries	380	24% aged 30–40 years, 33% aged 40–50 years, 29% aged 50–60 years; 16% France, 15% Spain, 12% Italy; 80% accredited gastroenterologists, 18% GI residents/fellows	AI in capsule endoscopy
Hah and Goldin (52)	Mixed methods	Clinicians having experience with patient diagnosis encounters using AI-based diagnostic technology, in the USA	114	66.7% aged 26–40 years; 84.2% female; 49.1% white; all bachelor’s degree or higher	AI in diagnostic decision making
Huisman et al. (33)	Quantitative	Radiologists and radiology residents from 54 countries, worldwide	1041	Median age 38 years (IQR 24–70); 34.3% female; 83% from Europe; 66% radiologists; 70% with no advanced scientific background (PhD or research fellowship)	AI in radiology
Martinho et al. (70)	Qualitative	Medical doctors (residents and specialists) from 13 different specialties including medical specialties (Family Medicine, Rheumatology, Dermatology, Intensive Medicine, Oncology, Neurology), surgical specialties (Surgery, Ophthalmology, OBGYN, Anesthesiology, Rehabilitation Medicine, Neurology), and diagnosis specialties (Pathology, Radiology/Nuclear Medicine/Neuroradiology) based in the Netherlands, Portugal and United States	77	Not reported	AI in medicine, broadly defined
Zheng et al. (26)	Quantitative	Medical workers and other professional technicians, mainly members of the Zhejiang Society of Mathematical Medicine, with locations covering various cities and counties mainly in Zhejiang Province, China	562	60.5% aged 25–45 years; 61.6% female; 51.8% medical workers; 66.4% bachelor’s degree or higher	AI in ophthalmology
Pumplun et al. (74)	Qualitative	Medical experts from clinics and their suppliers, location not reported	22	Mainly physicians with more than 3-year expertise	Machine Learning Systems for Medical Diagnostics
Park et al. (12)	Quantitative	Medical students, in the United States	156	25.8% in the first year of medical school, 27.1% in the second year	AI in medicine, broadly defined
Huisman et al. (34)	Quantitative	Radiologists and radiology residents from 54 countries, mostly Europe	1041	Median age 38 years (IQR 24–74); 35% female; 83% working in European countries; 66% radiologists, 35% residents	AI in radiology
Zhai et al. (66)	Quantitative	Radiation oncologists and medical students having clinical experience in using the computational system for contouring, from the Department of Radiation Oncology at Sun Yat-sen University Cancer Center, in China	307	87.6% aged 18–40 years; 50.8% female; all bachelor’s degree or higher	AI assisted contouring technology
Chen et al. (68)	Qualitative	Twelve radiologists and 6 radiographers from four breast units in NHS organizations and one focus group with eight radiographers from a fifth NHS breast unit, in the United Kingdom	26	Not reported	AI in radiology
Nelson et al. (64)	Quantitative	Dermatologist fellows of the AAD, in the United States	121	Mean age 51 years (SD 12); 47% female; 84% white; 95% non-Hispanic/Latino	AI in dermatology
Valikodath et al. (50)	Quantitative	Pediatric ophthalmologists who are members of AAPOS, in the United States	80	Mean age 21 years (range 0–46); 47% female	AI in ophthalmology
Kochhar et al. (35)	Quantitative	Physicians who are not currently involved with AI research in gastroenterology, location not reported	165	Not reported	AI in gastroenterology
Scheetz et al. (23)	Quantitative	Trainees and fellows of RANZCO, RANZCR, and ACD, in Australia and New Zealand	632	20.4% of RANZCO, 5.1% of RANZCR and 13.2% of ACD; 72.8% in metropolitan areas; 47.9% in practice for 20 years or more	AI in ophthalmology, dermatology, radiology and radiation oncology
Wong et al. (53)	Quantitative	Radiation oncologists, radiation therapists, medical physicists, and radiation trainees from 10 provinces, in Canada	159	Not reported	AI in radiation oncology
Layard Horsfall et al. (54)	Mixed methods	Surgical team (surgeons, anesthetists, nurses, and operating room practitioners), worldwide	133	31% aged 31–40 years; 30% female; 42% surgeons, 30% anesthetists	AI in neurosurgery
Cho et al. (36)	Quantitative	Medical students, in South Korea	100	Median age 22.5 years (range 19–37); 47% female	AI in dermatology
Yurdaisik and Aksoy (37)	Quantitative	Physicians, residents, and technicians working in radiology departments of various hospitals and medical students in Istinye university, in Turkey	204	81.8% aged 18–39 years; 59.8% female; 22.1% radiologists, 27.5% residents, 31.9% medical faculty students	AI in radiology
Qurashi et al. (21)	Quantitative	Radiologists, radiographers, clinical application specialists, and internship radiography students, in Saudi Arabia	224	75.9% aged <34 years; 38.4% female; 53.6% radiographers, 20.5% internship radiography students; 94.6% bachelor’s degree or higher	AI in radiology
Coppola et al. (55)	Quantitative	Radiologists who are members of SIRM, in Italy	1032	65.8% aged 36–65 years; 46.6% in non-academic hospitals	AI in radiology
Bisdas et al. (17)	Quantitative	Undergraduate medical and dental students across the world, worldwide	3133	Mean age 21.95 years (SD 2.77); 66.5% female; 26.43% in developed countries; 79.63% medical students	AI in medicine, broadly defined
Tran et al. (38)	Quantitative	Medical students from different provinces (Hanoi, Ho Chi Minh city, and other provinces), in Vietnam	211	Mean age 20.6 years (SD 1.5); 73.5% female; 89.1% in urban areas; 59.7% in Ho Chi Minh city; 57.8% general physicians	AI-based diagnosis support system
Wood et al. (51)	Quantitative	117 medical students and 44 clinical faculty from MCG, in the United States	161	Students: 52% aged ≤24 years; 45% female; 30% first-year, 29% second-year Faculty: 56% aged ≥50 years; 33% female	AI in medicine, broadly defined
Prakash and Das (67)	Mixed methods	Radiologists and doctors specialized in radiology and image, in India	104	82.51% aged <40 years; 36.07% female; 63.93% with 0–5-year experience; 57.92% resident radiologists and 34.97% consultant radiologists	Intelligent clinical diagnostic decision support systems
Staartjes et al. (75)	Quantitative	Neurosurgeons from EANS and CNS, worldwide	362	32.6% aged 30–40 years; 11.8% female; 67.4% in academic hospital; 69.1% in North America, 18.8% in Europe	Machine learning in neurosurgery
Batumalai et al. (47)	Quantitative	RT, MP, and RO from 93 radiotherapy treatment centers, in Australia	325	Majority born 1965–1995; all with >5 years practicing; 67.4% in Metropolitan place with public service (81.8%); 204 RTs, 84 MPs and 37 ROs	AI in radiation oncology, automation in radiotherapy planning
Polesie et al. (18)	Quantitative	Pathologists who regularly analyzed dermatopathology slides/images from 91 countries, worldwide	718	Median age 38 years (range 22–79); 64.1% females; 39.0% with access to WSI at work	AI in dermatopathology
Polesie et al. (19)	Quantitative	Dermatologists from 92 countries, worldwide	1271	Median age 46 years (IQR 37–56); 55.4% female; 69.8% working in Europe	AI in dermatology
Eltorai et al. (39)	Quantitative	Radiologists who are members of the Society of Thoracic Radiology and computer science experts from leading academic centers and societies, in the United States	95	Mean age of radiologists 52 years and mean age of computer scientists 45.5 years; 95 radiologists and 45 computer scientists; 78.9% of radiologists from university-based setting	AI in radiology
Petitgand et al. (76)	Qualitative	Healthcare managers, AI developers, physicians, and nurses, in Canada	30	Not reported	AI based decision support system in emergency care
Shen et al. (56)	Quantitative	Dermatologists from 30 provinces, autonomous regions, municipalities, and other regions (including Hong Kong, Macau, and Taiwan), in China	1228	Mean age 36.84 years (SD 8.86); 61.2% female; 89.5% bachelor’s degree or higher; 29.8% resident physicians, 38.5% attending physicians; 60.7% in tertiary hospitals	AI in dermatology
Petkus et al. (57)	Mixed methods	Specialty societies and committees, in the United Kingdom	18 medical specialty societies	Not reported	Clinical decision support systems (CDSS)
Doraiswamy et al. (63)	Quantitative	Psychiatrists from 22 countries in North and South America, Europe, and Asia-Pacific, worldwide	791	40% aged <44 years; 29.2% female; 64% white; 52% in public clinics	AI in psychiatry
Castagno and Khalifa (40)	Qualitative	Healthcare professionals (medical doctors, nurses, therapists, managers, and others), in the United Kingdom	98	34 medical doctors, 23 nurses, 11 managers, 7 therapists, and 23 other professionals	AI in medicine, broadly defined
Abdullah and Fakieh (58)	Quantitative	Healthcare employees (doctors, nurses, and technicians) at four of the largest hospitals in Riyadh, Saudi Arabia	250	74.4% aged 20–40 years; 74.8% female; 28% doctors, 48.4% nurses; 81.2% bachelor’s degree or higher	AI in medicine, broadly defined
Blease et al. (59)	Quantitative	Psychiatrists registered with Sermo, from 22 countries representing North America, South America, Europe, and Asia-Pacific, worldwide	791	61% aged >45 years; 29.2% female; 64.3% white; 52% in public clinics; 34.9% in the United States	AI in psychiatry
Wadhwa et al. (20)	Quantitative	Gastroenterologists (private practitioners, academic practice physicians, and gastroenterology fellows), in the United States	124	54.9% with >15 years of post-fellowship experience	AI in colonoscopic practice
Sit et al. (41)	Quantitative	Medical students with a valid United Kingdom medical school email address, in the United Kingdom	484	Not reported	AI in medicine, broadly defined
Bin Dahmash et al. (42)	Quantitative	Medical students in three different medical schools in Riyadh, Saudi Arabia	476	39.5% females	AI in radiology
Brandes et al. (43)	Quantitative	Medical students in different faculties of medicine in the city of São Paulo, Brazil	101	60% in the sixth year, 17% in the fifth year and 23% in the fourth year	AI in radiology
Kasetti and Botchu (60)	Quantitative	Medical students, in the United Kingdom	100	Not reported	AI in radiology
Sarwar et al. (11)	Quantitative	Pathologist-respondents practicing in 54 countries, worldwide	487	29.3% aged <35 years; 46.1% female; 49.6% practising pathologists, 25.5% residents/fellows; 24.9% Canada, 22.2% United States, and 10.5% United Kingdom	AI in pathology
Waymel et al. (25)	Quantitative	Radiologists (radiology residents and senior radiologists) registered in two departments, in France	270	Mean age 39.7 years (range 24–71, SD 12.3); 32.2% female	AI in radiology
Gong et al. (44)	Quantitative	Medical students in all 17 Canadian medical schools, in Canada	332	21.7% ranked radiology as the first specialty choice, 9% as the second choice, 10.6% as the third choice	AI in medicine, broadly defined
Pinto dos Santos et al. (45)	Quantitative	Undergraduate medical students, in Germany	263	Median age 23 years (IQR 21–26); 63.1% female	AI in medicine, broadly defined
Oh et al. (46)	Quantitative	Medical students, doctors who graduated from Soonchunhyang Medical College, and doctors at hospitals affiliated with Soonchunhyang University, in South Korea	669	22.4% aged <30 years; 22.1% female; 121 medical students, 162 training physicians, and 386 physicians	AI in medicine, broadly defined
Blease et al. (62)	Qualitative	General practitioners from all regions, in the United Kingdom	66	83% aged >45 years; 42% female; 55% part-time	AI in primary care
European Society of Radiology [ESR] (22)	Quantitative	Members of ESR, including radiologist, radiology residents, physicists, and engineers/computer scientists, in Europe	675	32.7% female; 94.1% radiologists; 82% in academic/public hospitals	AI in radiology
Pan et al. (65)	Mixed methods	Medical practitioners from five different hospitals in Anhui province, in China	484	75.61% aged <40 years; 45.45% female; 40.7% postgraduate education level; 60.12% <10 years work experience; 83.88% in large public hospital; 46.28% residents; 71.28% in clinical department	AI-driven smart healthcare services
van Hoek et al. (61)	Quantitative	Radiologists, students, and surgeons throughout the German speaking part, in Switzerland	170	40% female; 59 radiologists, 56 surgeons and 55 students	AI in radiology

ESPR, European Society of Pediatric Radiology; SPR, Society of Pediatric Radiology; ANZSPR, Australian and New Zealand Society for Pediatric Radiology; BMUS, British Medical Ultrasound Society; SoR, Society of Radiographers; NHS, National Health Services; AAD, American Academy of Dermatology; AAPOS, American Association for Pediatric Ophthalmology and Strabismus; RANZCO, Royal Australian and New Zealand College of Ophthalmologists; RANZCR, Royal Australian and New Zealand College of Radiologists; ACD, Australasian College of Dermatologists; SIRM, Society of Medical and Interventional Radiology; MCG, Medical College of Georgia; EANS, European Association of Neurosurgical Societies; CNS, Congress of Neurosurgeons; RT, Radiation Therapists; MP, Medical Physicists; RO, Radiation Oncologists; ESR, European Society of Radiology.

Of the 818 individuals who clicked on the link to our questionnaire, 13 did not give their consent to participate in the survey. Additionally, 47 responders were removed from further analysis because they did not meet the requirements of our target population or because they provided an inappropriate answer to the quality control question. Finally, 758 individuals from 39 countries completed the survey, of whom 96 (12.66%) were from low- and lower-middle-income countries. Geographic distribution of responders has also been provided in Figure 2. Table 2 provides details around the characteristics of our responder sample. The average age of respondents was 30.63 years. 532 (70.18%) respondents were women. 344 (45.38%) were practising physicians and the remaining 414 (54.62%) were medical students.

**TABLE 2:** Respondent characteristics of the questionnaire survey.

Variables	*N* (%)
Mean (SD) for age, year (*N* = 758)	30.63 (9.81)
Age, years (*N* = 758)	
<25	281 (37.07)
25–44	385 (50.79)
≥45	92 (12.14)
Gender (*N* = 758)	
Male	226 (29.82)
Female	532 (70.18)
Country income level (*N* = 758)	
Low- and lower-middle-income	96 (12.66)
High- and upper-middle-income	662 (87.34)
Identity (*N* = 758)	
Physician	344 (45.38)
Medical student	414 (54.62)
Education level (*N* = 344)*^†^	
Bachelor’s degree or below	188 (54.65)
Master’s or higher degree	156 (45.35)
Specialty (*N* = 344)*	
Internal medicine	16 (4.65)
Surgery	26 (7.56)
Obstetrics and gynecology	137 (39.83)
Pathology	95 (27.62)
Radiology or ultrasound	24 (6.98)
Other	46 (13.37)
Hospital level (*N* = 344)*	
Primary or secondary hospital	121 (35.17)
Tertiary hospital	223 (64.83)
Title (*N* = 344)*	
Resident physician	93 (27.03)
Attending physician	139 (40.41)
Associate chief or chief physician	112 (32.56)
Work experience (years) (*N* = 344)*	
≤10	152 (44.19)
>10	192 (55.81)
Learning stage (*N* = 414)**	
Undergraduate	231 (55.80)
Master or doctoral student	183 (44.20)
Major (*N* = 414)**	
Non-clinical medicine	159 (38.41)
Clinical medicine	255 (61.59)
Clinical practice experience (*N* = 414)**	
No	178 (43.00)
Yes	236 (57.00)

758 respondents were included in the analysis, of which 344 individuals were physicians and 414 individuals were medical students.

*Only 344 physicians were asked.

**Only 414 medical students were asked.

^†^Information of income level was extracted from the World Bank. New World Bank country classifications by income level: 2021-2022; Available from: https://blogs.worldbank.org/opendata/new-world-bank-country-classifications-income-level-2021-2022.

### Understanding and experience of clinical artificial intelligence

According to the systematic review, 5 (62.50%) out of eight included studies reported 65% or higher awareness of the wide application of clinical AI among physicians and medical students (16–20). Between 10–30% of all respondents had actually used clinical AI systems in their practice (18, 19, 21–27). This finding was consistent with the findings of our survey, with that only 148 (19.53%) participants having direct experience of clinical AI. We found that physicians were more likely to have used clinical AI than medical students (27.62% versus 12.80%, *p* < 0.001). Of those who had used AI systems, 103 (69.59%) indicated that they had encountered errors made by AI. 69 (46.62%) reported patient supportive attitude to clinical AI, but 30 (20.27%) were unclear about patient views. Detailed information is provided in Table 3.

**TABLE 3:** Respondent practical experience of clinical artificial intelligence (AI) over the past year.

Practice experience of clinical AI	Total (*n* = 758) *N* (%)	Physicians (*n* = 344) *N* (%)	Medical students (*n* = 414) *N* (%)	*p*-value*
Have used decision-support clinical AI systems in practice				<0.001
No	610 (80.47)	249 (72.38)	361 (87.20)	
Yes	148 (19.53)	95 (27.62)	53 (12.80)	
Use frequency**				0.263
Only once a year	20 (13.51)	12 (12.63)	8 (15.09)	
At least once every 6 months	25 (16.89)	13 (13.68)	12 (22.64)	
At least once a month	33 (22.30)	19 (20.00)	14 (26.42)	
At least once a week	35 (23.65)	24 (25.26)	11 (20.75)	
Every day	35 (23.65)	27 (28.42)	8 (15.09)	
Have met clinical AI error**				0.207
No	45 (30.41)	25 (26.32)	20 (37.74)	
Yes	103 (69.59)	70 (73.68)	33 (62.26)	
Patient attitudes toward clinical AI**				0.219
Oppose	2 (1.35)	1 (1.05)	1 (1.89)	
Neutral	47 (31.76)	25 (26.32)	22 (41.51)	
Support	69 (46.62)	48 (50.53)	21 (39.62)	
Unclear	30 (20.27)	21 (22.11)	9 (16.98)	

*Chi-square test.

**Only 148 respondents who have used decision-support clinical AI systems in the past year were asked.

**FIGURE 3:**
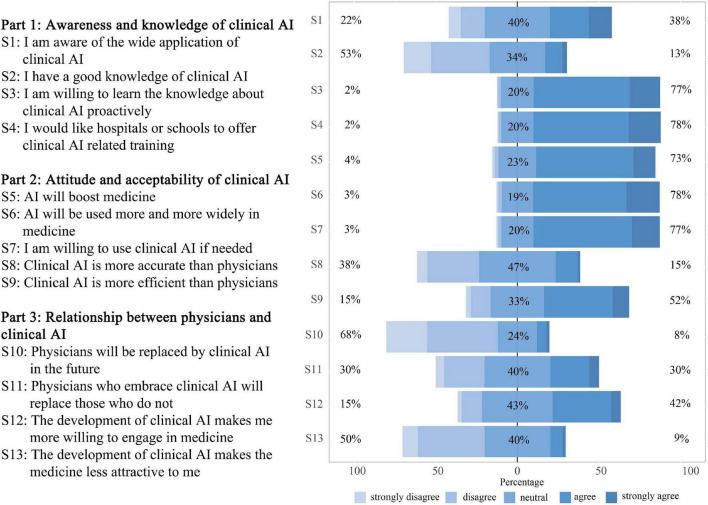
Respondent perspectives toward clinical artificial intelligence (AI). 13 statements were set to assess respondent perspectives toward clinical AI from three dimensions. Statement 1 to 4 assessed respondent awareness and knowledge of clinical AI. Statement 5 to 9 assessed attitude and acceptability of clinical AI. Statement 10 to 13 assessed respondent perception of the relationship between physicians and clinical AI.

Thirty-five included studies mentioned the knowledge level of physicians or medical students on clinical AI, of which 26 (74.29%) showed that participants lacked basic knowledge (16–19, 23, 25, 26, 28–46). Many physicians felt that the current training and educational tools, provided by their departments, were inadequate (47, 48). Medical students also felt that they mainly heard about AI from media and colleagues, but received minimal training from their schools (18, 30). Accordingly, 15 studies suggested an urgent need to integrate AI into residency programs or school curricula (17–19, 21, 29–33, 38, 41, 45, 49–51). Our questionnaire appears to confirm this situation with few respondents having good knowledge of AI (13% agreement). Our respondents also expressed a high willingness to learn (77% agreement) as well as a demand for relevant training to be provided by hospitals or schools (78% agreement). Please see Figure 3 for further details.

### Attitude and acceptability of clinical artificial intelligence

Forty-five included studies mentioned the views of physicians and medical students on clinical AI, and more than 60% of the respondents in 38 (84.44%) studies had an optimistic outlook regarding it (11, 12, 17–20, 22–26, 29, 30, 32, 33, 35, 36, 38–41, 45–61). For example, 75% of 487 pathologists from 59 countries were enthusiastic about the progress of AI (11); 77% of 1271 dermatologists from 92 countries agreed that AI would improve dermatologic practice (19). Similar positive opinions also existed among radiologists (22, 23, 25, 29, 33, 39, 47, 48, 53, 55, 61), gastroenterologists (24, 35), general practitioners (28, 62), psychiatrists (59, 63), ophthalmologists (23, 50). Additionally, in 14 studies reporting use intentionality, more than 60% respondents in 10 (71.43%) studies were willing to incorporate AI into their clinical practice (17, 21, 26, 34, 36, 44, 49, 55, 56, 61). The perceived benefits of AI included promoting workflow efficiency, quality assurance, improving standardization in the interpretation of results, as well as liberating doctors from mundane tasks and providing more time to expand their medical knowledge and focus on interacting with patients (11, 22, 35, 50, 64). Participants in our survey were also optimistic about the prospect of clinical AI and showed a high intention of use, with 78% in agreement that “AI will be used more and more widely in medicine” and 77% agreed that they are “willing to use clinical AI if needed” (Figure 3).

Although participants in several studies, included in the systematic review, believed that AI diagnostic performance was comparable and even superior to human doctors (3, 37, 46, 52), many respondents expressed a lack of trust in clinical AI and preferred results checked by human clinicians, and voiced concerns about the unpredictability of results and errors related to clinical AI (11, 33, 45, 48). Other concerns mentioned included operator dependence and increased procedural time caused by clinical AI, poor performance of AI in unexpected situations, and its lack of empathy or communication (20, 46, 62). In our questionnaire, few agreed that AI is more accurate than physicians (15% agreement), but these objectors seemed to be more confident in AI’s efficiency with 52% agreeing that “clinical AI is more efficient than physicians” (Figure 3).

**FIGURE 4:**
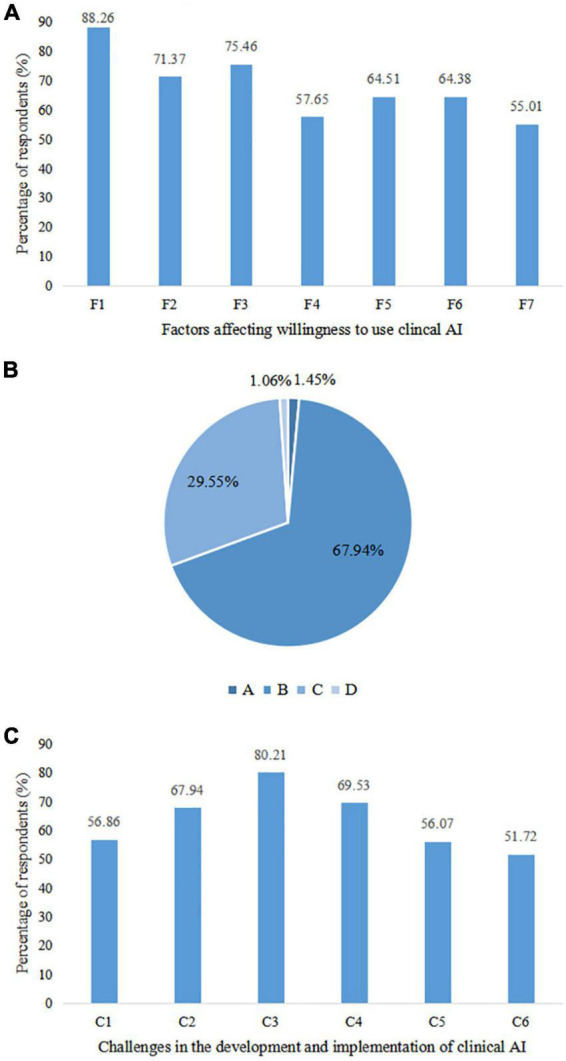
Factors related to use willingness, perceived relationship between physicians and artificial intelligence (AI), and challenges faced by clinical artificial intelligence (AI). **(A)** Factors associated with willingness to use clinical AI. F1: Accuracy; F2: Efficiency; F3: Ease of use; F4: Widely adopted; F5: Cost-effectiveness; F6: Interpretability; F7: Privacy protection capability. **(B)** Perceived relationship between physicians and clinical AI. A: Physicians don’t need to use clinical AI; B: Physicians lead the diagnosis and treatment process while clinical AI only plays an auxiliary role; C: Clinical AI completes the diagnosis and treatment process independently under the supervision and optimization of physicians; D: Clinical AI completely replaces physicians for diagnosis and treatment. **(C)** Challenges to be overcome in the development and implementation of clinical AI. C1: Inadequate algorithms and computational power of clinical AI; C2: Lack of high-quality data for clinical AI training; C3: Lack of inter-disciplinary talents with both medical and AI knowledge; C4: Lack of regulatory standards; C5: Difficulties in integrating clinical AI with existing medical process; C6: Insufficient understanding and acceptance of clinical AI among physicians and medical students.

Four studies used structural equation modeling to identify determinants of adoption intention for clinical AI among healthcare providers and medical students (38, 65–67). Perceived usefulness, the experience of using mHealth, subjective norms, and social influence had a positive effect on adoption intention, while perceived risk had the opposite effect. In our questionnaire, accuracy, ease of use, and efficiency were the top three perceived factors affecting respondent willingness to use clinical AI, with more than 70% considering these elements. Cost-effectiveness and interpretability followed, with more than 60% voicing their concerns (Figure 4A).

### Relationship between physicians and clinical artificial intelligence

Forty included studies mentioned potentially replacing physicians and changes in employment market caused by clinical AI. The support rate for the statement that AI could replace human physicians ranged from 6 to 78% (19, 37, 58), of which 31 (77.50%) studies showed that the support rate was less than half (11, 16–19, 21, 22, 24, 25, 30, 33–37, 39–42, 44–50, 55, 56, 59, 60, 63). Radiologists did not view AI as a threat to their professional roles or their autonomy, however, radiographers showed greater concern about AI undermining their job security (68). In our questionnaire, most disagreed that physicians will be replaced by AI in the future (68% disagreed). Although the number of those in agreement and with disagreement was balanced around whether physicians who embrace AI will replace those who do not (30% agreement vs. 30% disagreement; Figure 3).

In spite of the controversial opinions, there was consensus that AI should become a partner of physicians rather than a competitor (17). Respondents from several studies predicted that humans and machines would increasingly collaborate on healthcare (11, 17, 56, 59, 69). However, diagnostic decision-making should remain a predominantly human task or one shared equally with AI (11), which was consistent with our findings, that 68% agreed that AI should assist physicians (Figure 4B). While AI can assist in daily healthcare activities and contribute to workflow optimization (33, 56), physicians were not comfortable acting on reports independently issued by AI, and double checking by physicians would be preferred (39, 69). All investigated members of the European Society of Radiology believed that radiologists should be involved in clinical AI development and validation. 434 (64%) thought that acting as supervisors in AI projects would be most welcomed by radiologists, followed by 5359 (3%) who considered task definition and 197 (29%) in image labeling (22). Respondents from 18 medical societies and committees also pointed out that involving physicians in system design, procurement and updating could help realize the benefits of clinical decision support systems (57).

Clinical AI was considered as an influencer behind career choices, and radiologists seemed to be the most affected specialty with almost half of all medical students feeling less enthusiastic about their specialty as a result of AI (27, 34, 39, 41–44, 61). Yurdaisik et al. reported 55% of their sample of respondents thought that new physicians should choose professional fields in which AI would not dominate (37). However, developments in AI also positively affected career preferences for many physicians and medical students, making them optimistic about the future in their chosen specialty (25, 36, 37). Our survey found that 42% believed that the development of clinical AI made them more willing to engage in medicine, although 9% reported that it actually made medicine a less attractive option (Figure 3).

### Challenges to clinical artificial intelligence development and implementation

Multiple challenges were emphasized in the development and implementation of clinical AI, including an absence of ethically defensible laws and policies (11, 33, 49, 55, 57, 59), ambiguous medico-legal responsibility for errors made by AI (11, 22–24, 37, 48, 57), data security and the risk of privacy disclosure (35, 40, 54, 69), “black box” nature of AI algorithms (57, 70), low availability of high-quality datasets for training and validation (57), and shortage of interdisciplinary talents (11). Among the respondents in our survey, the lack of interdisciplinary talents was the primary concern, followed by an absence of regulatory standards and a scarcity in high-quality data for AI training (Figure 4C).

### Statistically significant associations

**FIGURE 5:**
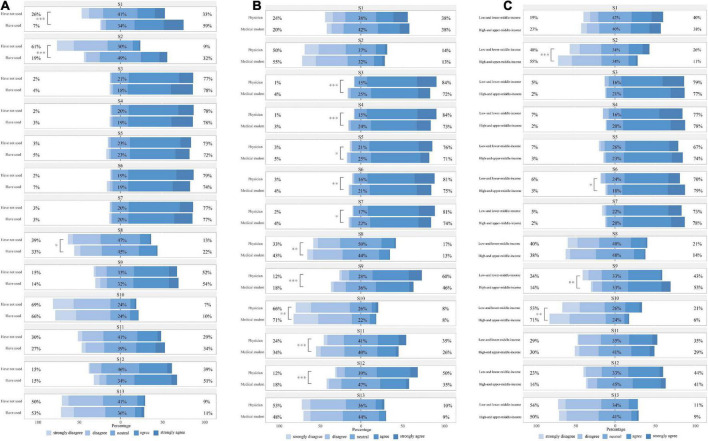
Subgroup analysis of responses to 13 statements. **(A)** By clinical artificial intelligence (AI) use experience; **(B)** By identity; **(C)** By country specific income levels. Mann–Whitney *U* test, *p < 0.05, **p < 0.01, ***p < 0.001.

A comparison of response distributions across subgroups has been provided in Figure 5 and Supplementary Table 2. Moreover, Figure 5A illustrates that respondents who have used clinical AI in the past year expressed stronger feelings about the wide application of AI and reported having a better understanding of AI-related knowledge than those who had not. They were also more positive when considering the accuracy of clinical AI technologies. As can be seen in Figure 5B, in general, where there was a statistically significant difference between identities, physicians carried a more optimistic outlook regarding the performance and prospect of clinical AI, and expressed stronger willingness to use and learn clinical AI. Physicians also agreed more than medical students, that physicians would be replaced by clinical AI and conservative physicians will be replaced by those who embrace AI. Facing the rapid development of clinical AI, physicians showed greater enthusiasm than medical students.

Figure 5C compares respondent views on clinical AI in countries with different income levels. Compared with respondents from high- and upper-middle-income countries, those from low- and lower-middle-income countries reported subjectively more knowledge around AI, but tended to be less confident about the efficiency and wide application of clinical AI, with more agreeing that AI would replace physicians. Multivariable logistic regression revealed that physicians who worked in tertiary hospitals were more willing to use clinical AI [aOR 2.16 (1.11–4.25)]. Older physicians were also more positive about using clinical AI [aOR 1.08 (1.02–1.16)]. There were no statistically significant differences between medical students from various backgrounds. Detailed information has been provided in the Supplementary Tables 3, 4.

## Discussion

Through this systematic review and evidence-based survey, we found that most physicians and medical students were aware of the increasing application of AI in medicine. However, few had actually experienced clinical AI first-hand and there appears to be a lack basic knowledge about these technologies. Overall, participants appeared optimistic about clinical AI but also had reservations. These reservations were not entirely dependent upon AI performance, but also appear related to responder characteristics. Even though the notion that AI could replace human physicians was contended, most believed that the collaboration between the two should be strengthened while maintaining physician’s autonomy. Additionally, a number of challenges emerged regarding clinical AI development pathways and around implementing novel AI technologies.

There is an optimistic yet reserved attitude about clinical AI, which suggests that AI is widely considered a complex socio-technical system with both positive and negative aspects. Rather than the physician spending a lot of time analyzing a patient’s condition in real-time, AI can process a huge amount clinical data using complex algorithms, which can provide diagnosis and treatment recommendations more quickly and more accurately (46, 58, 62). Although, it is also held that AI can generate unpredictable errors in uncommon or complex situations, especially where there is no specific algorithmic training (11). Actually, since the data sets used to train AI models always appear to exclude elderly people, rural communities, ethnic minorities, and other disadvantage groups, AI’s outputs might be inaccurate when applied to under-represented populations (6). Another issue in establishing trust in AI is the poor interpretability of AI algorithms. To be fair, algorithms with good explainability and high accuracy cannot be developed overnight. Therefore, it is particularly important to clearly explain the validation process of AI systems. Physicians need more information, such as data used for AI training, model construction process, and variables underlying AI models, to help them judge whether the AI results are reliable. However, unclear methodological interpretation, lacking a standardized nomenclature and heterogeneity in outcome measures for current clinical research limits the downstream evaluation of these technologies and their potential real-word benefits. Considering issues raised by AI-driven modalities, many well-known reporting guidelines have been extended to AI versions to improve reproducibility and transparency of clinical studies (71, 72). However, it takes time to establish norms and then to generate high-quality research outputs.

Although the current discourse around physician acceptance and utility of clinical AI has shifted from direct replacement to implementation and incorporation, the adoption of AI still has the possibility of transferring decision-making from human to machines, which may undermine human authority. In order to maintain autonomy in practice, physicians need to learn how to operate AI tools, judge the reliability of AI results outputs, as well as redesign current workflows. It appears that the most adaptable physicians, those who embrace AI will progress, while those who are unable or unwilling to adopt novel AI technologies may be left behind. Furthermore, physicians should not only become primary AI users, but also should be involved in the construction of AI technologies. The development of AI requires interdisciplinary collaboration, not just the task of computer scientists. Physicians have particular insight into clinical practice which can inspire AI developers to design AI tools that truly meet clinical needs. Physicians can also participate in the validation of AI systems to promote quality control.

Compared with the more positive views of direct clinical AI users, respondents without having had direct experience appeared to perceive clinical AI in more abstract manner and were more guarded in their opinions. Similarly, medical students appear to hold more conservative attitudes than physicians although this is at least partly due to limited experience. Physicians working in high-level hospitals are more likely to accept clinical AI than those from relatively low-level hospitals. This may be because there are differences in hospital resources which has influenced thinking about advancements in both superior and relatively inferior hospitals. High-level hospitals certainly have greater financial support with well-developed management mechanisms. Therefore, it might be wise to establish pilot AI programs in these hospitals. This will enable us to explore evolving practices and the challenges related to change, such as formulating new regulatory standards, defining responsibilities and determining accountability. Ensuring “early experiences” are captured and appraised will bring broader benefits to the community.

Our online questionnaire investigated some participants from low- and lower-middle-income countries who were not covered in previous studies. It was found that they were less optimistic about the prospect of clinical AI and more believed that AI would replace physicians than those from high- and upper-middle-income countries. Bisdas et al. also found that compared with medical students from developed countries, those from developing countries agreed less that AI will revolutionize medicine and more agreed that physicians would be replaced by AI (17). This discrepancy may be due to the gap in health infrastructures and in health workforces between countries with different income levels. For example, computed tomography (CT) scanner density in low-income countries is 1 in 65 of those in high-income countries (73). Having a Picture Archiving and Communication System (PACS) is also not so commonplace in low-income countries. However, many AI systems are embedded within hardware like CT scanners and are deployed using delivery platforms such as PACS. Therefore, inadequate infrastructures have seriously hampered the delivery and maintenance of AI. As for health workforce, skilled physicians in developed counties have the capability to judge AI outputs based on knowledge and clinical scenarios, but such expertise and labor are lacking in poorly resourced countries. Physicians in low-income countries may be less confident in their medical skills and may rely too much on AI, giving reason for the common belief that physicians will be replaced by AI. What we can say, is that the introduction of AI into resource-poor countries will proceed differently to high-income countries. Low-income countries need a site-specific tailored approach for integrating digital infrastructures and for clinical education, to maximize the benefits of clinical AI.

Before providing recommendations, we must acknowledge the limitations of this study. First, we did not assess risk of bias of each included study in the systematic review. We also note that our questionnaire and many of the studies included in the systematic review were Internet-based, which may have introduced non-response bias. The possibility that respondents are more likely to hold stronger views on this issue than non-respondents should be considered. Second, the relatively small sample size and uneven population distribution of our cross-sectional study means that our findings are less generalizable. Although we conducted subgroup analysis to evaluate differences in perspective among our respondents, these differences are likely to be fluid and to change as technologies evolve. However, the two-stage approach made our insights and comparisons more reliable. While beyond the remit of this study, we can see the general demand for AI-related education to overcome some of the anxieties associated with adopting new clinical AI technologies. Clearly, there is a need to incorporate health informatics, computer science and statistics into medical school and residency programs. This will increase awareness which can alleviate some of the stress involved in change, as well as facilitate safe and efficient implementation of clinical AI.

## Conclusion

This novel study combined a systematic review with a cross-sectional survey to comprehensively understand physician and medical student acceptance of clinical AI. We found that a majority of physicians and medical students were aware of the increasing application of AI in medicine, but most had not actually used clinical AI and lacked basic knowledge. In general, participants were optimistic about clinical AI but had reservations. In spite of the contentious opinions around clinical AI becoming a surrogate physician, there was unanimity regarding strengthening collaborations between AI and human physicians. Relevant education is needed to overcome potential anxieties associated with adopting new technologies and to facilitate the successful implementation of clinical AI.

## Data availability statement

The raw data supporting the conclusions of this article will be made available by the authors, without undue reservation.

## Ethics statement

The research ethics committee of the Chinese Academy of Medical Sciences and Peking Union Medical College approved this study (CAMS&PUMC-IEC-2022-022). Participation in the questionnaire was voluntary and informed consent was obtained before completing the questionnaire.

## Author contributions

MC, BZ, and PX conceptualized the study. BZ, ZC, and MC designed the systematic review, extracted data, and synthesis results. MC, BZ, MJG, NMA, and RR designed the questionnaire and conducted the analysis. MC and SS wrote the manuscript. YQ, PX, and YJ revised the manuscript. MC and BZ contributed equally to this article. All authors approved the final version of the manuscript and take accountability for all aspects of the work.
