# Supplementary material for: Acceptance of clinical artificial intelligence among physicians and medical students: A systematic review with cross-sectional survey
Source: Front Med (Lausanne). 2022 Aug 31;9:990604. doi: 10.3389/fmed.2022.990604 (PMC9472134; doi:10.3389/fmed.2022.990604)
Supplement: Supplementary file 1 [file Data_Sheet_1.pdf]

Search strategies of the systematic review

A) PubMed (1570 results, 6th March, 2022)
(((("artificial intelligence" OR "machine learning" OR "deep learning") AND (doctors OR physicians OR clinicians OR "health care providers" OR "health care professionals" OR "health care workers" OR "medical professionals" OR "medical workers" OR "health professionals" OR "health workers" OR "medical students" OR "health care students") AND ("Attitude to Computers"[Mesh] OR attitudes OR views OR opinions OR perspectives OR perceptions OR beliefs OR acceptability OR confidence OR adoption OR think)) NOT (Review[Publication Type])) NOT (Systematic Review[Publication Type])) NOT (Meta-Analysis[Publication Type]) AND ((english[Filter]) AND (2017:2022[pdat]))

B) EMBASE (1079 results, 6th March, 2022)
('artificial intelligence'/exp OR 'artificial intelligence' OR 'machine learning'/exp OR 'machine learning' OR 'deep learning'/exp OR 'deep learning') AND (doctor? OR 'physician'/exp OR physician? OR clinician? OR 'health care providers' OR 'health care professionals' OR 'health care workers' OR 'medical professionals' OR 'medical workers' OR 'health professionals' OR 'health workers' OR 'medical students' OR 'health care students') AND ('attitudes'/exp OR attitude? OR view? OR opinion? OR perspective? OR 'perceptions'/exp OR perception? OR 'beliefs'/exp OR 'beliefs' OR 'acceptability'/exp OR acceptability OR confidence OR adoption OR think) AND [2017-2022]/py AND [english]/lim NOT [review]/lim NOT [conference abstract]/lim

C) IEEE Xplore (516 results, 6th March, 2022)
Filter to exclude articles before 2017
("artificial intelligence" OR "machine learning" OR "deep learning") AND (doctors OR physicians OR clinicians OR “health care providers” OR “health care professionals” OR “health care workers” OR “medical professionals” OR “medical workers” OR “health professionals” OR “health workers” OR “medical students” OR “health care students”) AND (attitudes OR views OR opinions OR perspectives OR perceptions OR beliefs OR acceptability OR confidence OR adoption OR think)

D) Web of Science (1614 results, 6th March, 2022)
Topic search
((TS=(("artificial intelligence" OR "machine learning" OR "deep learning") AND (doctors OR physicians OR clinicians OR “health care providers” OR “health care professionals” OR “health care workers” OR “medical professionals” OR “medical workers” OR “health professionals” OR “health workers” OR “medical students” OR “health care students”) AND (attitudes OR views OR opinions OR perspectives OR perceptions OR beliefs OR acceptability OR confidence OR adoption OR think))) AND (PY==("2022" OR "2021" OR "2020" OR "2019" OR "2018" OR "2017")) AND (LA==("ENGLISH"))) NOT (DT==("REVIEW"))
