# Supplementary material for: Acceptance of clinical artificial intelligence among physicians and medical students: A systematic review with cross-sectional survey
Source: Front Med (Lausanne). 2022 Aug 31;9:990604. doi: 10.3389/fmed.2022.990604 (PMC9472134; doi:10.3389/fmed.2022.990604)
Supplement: Supplementary file 2 [file Data_Sheet_2.pdf]

Questionnaire of the survey on acceptance of clinical AI among physicians and medical students

Dear participants,

We are a research team from Peking Union Medical College and are inviting you to join our study. Before you decide whether to participate, please read the following first.

Artificial Intelligence (AI) is a new technical science that studies and develops theories, methods, technologies and application systems for simulating, extending and expanding human intelligence. As the increasing application of AI in health care, we are conducting this survey to understand the attitudes and perceptions of medical students and physicians toward clinical AI. In this study, clinical AI is defined as **AI designed to automate intelligent behaviors in clinical settings for the purpose of supporting physician-mediated care-related tasks**, excluding specifically consumer-facing products like wearable devices. This survey will take approximately 5-10 minutes to complete. Your responses will be anonymous and kept strictly confidential. The results will be used for scientific research only. This survey is voluntary and you have the right to choose to participate or not.

Thanks for your cooperation and support!

1. Informed consent

○ I agree to take part in this survey

○ I don’t agree to take part in this survey [end survey]

2. Your identity

○ Physician

○ Medical student

○ Others [end survey]

3. Your gender

○ male ○ female

4. Your age (years old)

____

5. Your nationality

____

(Only physicians answer question 6 to 10)

6. Your education level

○ Bachelor's degree or below

○ Master's or higher degree

7. The level of your hospital

○ Primary hospital

○ Secondary hospital

○ Tertiary hospital

8. Your professional title

○ Resident

○ Attending physician

○ Associate chief physician

○ Chief physician

9. Your working experience (since resident)
○ ≤5 years
○ 6-10 years
○ 11-15 years
○ >15 years

10. Your specialty
○ Internal medicine
○ Surgery
○ Obstetrics and gynecology
○ Pathology
○ Radiology or ultrasound
○ Others, please specify __________________

(Only medical students answer question 11 to 13)

11. Your Major
○ Clinical medicine
○ Non-clinical medicine

12. Your current learning stage
○ Undergraduate
○ Master student
○ Doctoral student

13. Have you ever had clinical practice experience?
○ Yes
○ No

14. In the past year, have you ever used decision-support clinical AI systems in practice?
○ Yes
○ No

15. In the past year, how often have you used decision-support clinical AI systems in practice?
○ Only once a year
○ At least once every six months
○ At least once a month
○ At least once a week
○ Every day

16. Have there been any errors or accidents while working with decision-support clinical AI systems?
○ Yes

○ No

17. What are patients’ attitudes towards the use of decision-support clinical AI systems?
○ Oppose
○ Neutral
○ Support
○ Unclear

18. Do you agree with the following statements?

Statement	Strongly disagree	Disagree	Neutral	Agree	Strongly agree
I am aware of the wide application of clinical AI	○	○	○	○	○
I have a good knowledge of clinical AI	○	○	○	○	○
I am willing to learn the knowledge about clinical AI proactively	○	○	○	○	○
I would like hospitals or schools to offer clinical AI related training	○	○	○	○	○
AI will boost medicine	○	○	○	○	○
AI will be used more and more widely in medicine	○	○	○	○	○
I am willing to use clinical AI if needed	○	○	○	○	○
The development of clinical AI will cause many doctors to lose their jobs. This is a quality control question. No matter what you think, please choose disagree	○	○	○	○	○
Clinical AI is more accurate than physicians	○	○	○	○	○
Clinical AI is more efficient than physicians	○	○	○	○	○
Physicians will be replaced by clinical AI in the future	○	○	○	○	○
Physicians who embrace clinical AI will replace those who do not	○	○	○	○	○
The development of clinical AI makes me more willing to engage in medicine	○	○	○	○	○

The development of clinical AI makes the medicine less attractive to me	○	○	○	○	○

19. Which of the following factors will influence your willingness to use clinical AI? (there could be more than one option)

□ Whether it is reliable and accurate

□ Whether it is efficient and fast

□ Whether it is easy to use

□ Whether it is widely adopted

□ Whether it is cost-effective

□ Whether its decision-making process is interpretable

□ Whether it is privacy-protective

□ Others, please specify __________________

20. Which of the following challenges do you think clinical AI is facing? (there could be more than one option)

□ Inadequate algorithms and computational power of clinical AI

□ Lack of high-quality data for clinical AI training

□ Lack of inter-disciplinary talents with both medical and AI knowledge

□ Lack of regulatory standards

□ Difficulties in integrating clinical AI into existing medical process

□ Insufficient understanding and acceptance of clinical AI among physicians and medical students

□ Others, please specify __________________

21. How do you think physicians should work with clinical AI?

○ Physicians don't need to use clinical AI

○ Physicians lead the diagnosis and treatment process while clinical AI only plays an auxiliary role

○ Clinical AI completes the diagnosis and treatment process independently under the supervision and optimization of physicians

○ Clinical AI completely replaces physicians for diagnosis and treatment

Thank you for your participation!
