# Supplementary material for: Acceptance of clinical artificial intelligence among physicians and medical students: A systematic review with cross-sectional survey
Source: Front Med (Lausanne). 2022 Aug 31;9:990604. doi: 10.3389/fmed.2022.990604 (PMC9472134; doi:10.3389/fmed.2022.990604)
Supplement: Supplementary file 3 [file Data_Sheet_3.pdf]

Supplementary Table 1: Main findings of studies included in the systematic review

Author (year)	Understanding and experience of clinical AI	Attitude and acceptability of clinical AI	Relationship between physicians and clinical AI
Shelmerdine et al (2022) [1]	The majority of respondents do not use AI tools in daily practice (61.3%) or not sure (11.3%). Half (50.8%) did not report any knowledge of computer coding or programming.	Imaging health care professionals working with children had a positive outlook regarding the use of AI in paediatric radiology. About 63.3% of respondents believed the benefits of using AI in paediatric radiology either slightly or greatly outweighed any potential risks. 41.6% of respondents disagreed that AI would outperform paediatric radiologists for disease detection in the next decade.	The majority (85.4%) did not believe jobs in paediatric radiology would be replaced by AI.
Buck et al (2022) [2]	Most participants had poor AI literacy in the data set and had not yet interacted with AI-enabled systems.	This paper identified 5 determinants of GP's attitudes towards clinical AI, including concerns, expectations, environmental influences, and individual characteristics.	Half (50%) of the participants expressed existential anxiety connected with AI-enabled systems as they perceive that this technology can take over some of their tasks.
Abuzaid et al (2022) [3]	About 66% of the participants were excited and aware of AI. But 68.6% indicated that radiology workers lack a basic understanding of AI.	There was 85.6% disagreement with the statement that AI can play an important role in radiology.	About 64.1% thought that AI would not threaten or disrupt some professional radiology careers.
Khafaji et al (2022) [4]	Less than half (41.6%) of residents reported being familiar with AI. About 77.9% were willing to learn and train on ML algorithm so it could perform some of the tasks they do as radiologists and 76% wanted AI to be a part of residency training.	81.8% of participants agreed that AI would improve medicine in general.	43.5% of participants did not expect AI to affect job positions and 42% anticipated that job positions will decrease.
Lim et al (2022) [5]	NR	NR	If AI was to be integrated into clinical practice, 89.8% of referring doctors preferred a hybrid model over AI alone.
Kansal et al (2022) [6]	Most of the respondents (73.6%) either felt extremely unknowledgeable or unknowledgeable about the basic principles of	The majority (74.4%) felt that AI will play an important role in delivering healthcare services in the future.	76.4% either strongly disagreed or disagreed with the statement "Artificial Intelligence may replace physicians in some specialties in the

	AI and its applications in healthcare. 59% either strongly agreed or agreed with the statement “Formal training is needed in medical schools and hospitals to teach about Artificial Intelligence and its applications in healthcare.”		future.”
Eiroa et al (2022) [7]	There is a lack of knowledge about AI among Spanish radiologists. All the respondents consider that they should pursue academic training in medical informatics and new technologies, and 92.9% of them reckon this preparation should be incorporated in the training program of the specialty.	NR	Job loss was not seen as a peril by 45.7% of the participants.
Reeder et al (2022) [8]	While 56% of the respondents indicated a good understanding of radiology, only 30% of them did so for use of AI in radiology.	AI significantly lowered students' preference for ranking radiology.	AI has a significantly negative impact on US medical students' choice of radiology as a career. 40% of students expressed a concern towards choosing radiology due to AI, and 51% of students predicted a decrease in radiology job opportunities due to AI.
Teng et al (2022) [9]	When asked to define AI, more than half of the respondents (51.08%) did not know what AI was. 63.36% believed that gaining basic literacy in AI should be part of their curriculum.	74.5% reported a positive outlook toward the emerging role of AI in their respective fields. Attitudes toward AI varied by discipline.	78.77% predicted that AI technology would affect their careers within the coming decade
Pangti et al (2021) [10]	55.1% either strongly agreed or agreed with the statement “Artificial intelligence should be made part of training during dermatology residency.”	71% either strongly agreed or agreed with the statement “Artificial intelligence will benefit medicine in general.” and 61.3% agreed or strongly agreed with the statement “I am open to using artificial intelligence in my dermatology practice in the near future.”	12.6% felt that artificial intelligence will replace dermatologists in the future.
Leenhardt et al (2021) [11]	45% were aware of using any AI applications. But 38% did not consider themselves as well informed about the use of new technologies and 46% did not already use endoscopy AI solutions	80% agreed or strongly agreed that AI will positively impact CE.	71% at least moderately disagreed with the idea that AI would replace them at work.

	in their regular practice. 75% were interested or strongly interested in receiving a generic/baseline training on AI.		
Hah et al (2021) [12]	NR	Their overall sentiment toward AI-assisted diagnosis was positive and comparable with diagnoses made without the assistance of AI.	NR
Huisman et al (2021) [13]	57% were lack of knowledge. A majority (79%) indicated that AI should be incorporated in residency programs.	Most (89%) thought that AI will help to improve diagnostic radiology and 85% thought that AI will alter the future of radiologists. Only 37% reported lack of trust in AI.	47% thought radiologists would be partially replaced.
Martinho et al (2021) [14]	NR	Four main perspectives have emerged from the data representing different views about the ethics surrounding Health AI. "AI is a helpful tool" highlights the efficiency associated with automation; "Rules & Regulations are crucial" shows strong distrust in private tech companies and emphasizes the need for regulatory oversight; "Ethics is enough" puts more trust in private tech companies and maintains that ethics is sufficient to ground these corporations; "Explainable AI tools" emphasizes the importance of explainability of AI tools in order to ensure that doctors are engaged in the technological progress.	NR
Zheng et al (2021) [14]	69.4% had no related experience with AI in ophthalmology. About 1/3 of the respondents understood AI and ophthalmic AI. 43.6 % had related applications experience in ophthalmic AI.	59.1 % of the respondents had a relatively high acceptance level of AI in ophthalmology. 62.1% thought that the current development of AI in ophthalmology was good or very good.	About 77 % of the respondents believed that ophthalmic AI would completely or partially replace doctors.
Pumplun et al (2021) [15]	NR	Developed an integrative framework for the adoption process of machine learning systems in clinics, including technology, organization, wider system,	NR

		adopter system, condition, value proportion, and patient data.	
Park et al (2021) [16]	NR	Over 75% agreed that AI would have a significant role in the future of medicine.	Nearly half (44%) reported that AI made them less enthusiastic about radiology.
Huisman et al (2021) [18]	A minority of respondents (21%) had only heard of AI and a minority of respondents (16%) had advanced knowledge or were actively engaged in research and/or development of AI.	A positive attitude towards AI was observed in 48% and 85% were willing to use AI in clinical practice.	Fear of replacement was found in 38%. 79% agreed that radiologists should take the lead in development of AI technology. 8% indicated that they would have chosen a career as a radiologist again and 15% not.
Zhai et al (2021) [19]	NR	A model of AI-assisted contouring technology acceptance was developed. Behavioral intention was significantly affected by performance expectancy, social influence, and facilitating conditions. Effort expectancy, perceived risk, and resistance bias did not significantly affect behavioral intention.	NR
Chen et al (2021) [20]	We found that radiographers and radiologists vary with respect to their awareness and knowledge around AI. Radiologists receive more information and acquire more knowledge of the potential applications of AI. Radiographers instead rely more on localized personal networks for information.	NR	Radiologists are less concerned that AI technology might constrain their professional role and autonomy. Radiographers showed greater concern about the potential impact that AI technology could have on their roles and skills development.
Nelson et al (2021) [21]	NR	Only 46% thought AI would positively influence their practice.	NR
Valikodath et al (2021) [22]	91% reported understanding the concept of AI and 71% believed AI should be incorporated into medical school and residency curricula.	70% believed AI will improve the practice of ophthalmology and 68% reported willingness to incorporate AI into their clinical practice.	65% did not believe AI will replace physicians.
Kochhar et al	Most respondents (61.2%) who were unfamiliar	Most respondents were enthusiastic about the	37.1% believed that AI had the potential to

(2021) [23]	with AI were unsure of how to start an AI-related study.	progression of AI: 68.9% predicted it will improve healthcare quality and 61.2% predicted it will improve efficiency.	replace physicians at some point in the future.
Scheetz et al (2021) [24]	Respondents indicated that they had never used AI applications in their work as a clinician (80.9%). Almost half of respondents (47.6%) rated their knowledge of AI as average relative to their peers, with few rating their knowledge as excellent (5.5%) or very poor (4.9%).	The majority (71%) believed artificial intelligence would improve their field of medicine. Most survey respondents considered that AI systems would need to achieve performance that was superior to the average performing specialist when applied to screening for disease (64.1%) or for diagnostic decision support (80.1%).	The majority of respondents (71.1%) reported that AI will impact workforce needs 'somewhat' or 'to a great extent' in medicine within the next decade and 85.8% believed that beyond the next decade.
Wong et al (2021) [25]	Knowledge about AI was moderate with an average of 5/10, but 91% responded interest in learning more about it.	Radiation oncology professionals believe AI will be an important part of patient treatment in their future practices.	NR
Layard Horsfall et al (2021) [26]	33% found AI useful but stressed the importance of first understanding its limitations.	The majority of surgeons and the wider surgical team both agree and are comfortable with the application of AI within neurosurgery. 36% had no prior AI research experience but expressed interest in using AI in their own practice.	NR
Cho et al (2021) [27]	10% stated that they know AI well, whereas 42% stated they do not. 22% were aware that AI can be used to diagnose dermatological diseases.	95% agreed that AI will be used in dermatology and 84% said that they will use AI when they become medical doctors.	Only 6% agreed that AI will replace the dermatologists. When considering the potential changes to dermatology that can be led by developments in AI, 50% stated that this has positively affected their preference, 12.5% stated that this has negatively affected their preference, and 31.25% stated no influence on their preference.
Yurdaisik et al (2021) [28]	47.1% of the participants reported that they have enough knowledge about AI applications in general,	22.5% believed that in establishing a diagnosis AI will be superior over radiologists in near future.	The rate of participants who thought that AI will completely replace radiologists was only 6.3%. Among the participants, 54.9% thought

	while only 25% stated that they had sufficient knowledge about AI applications in radiology.		that new physician candidates should choose specialty areas where AI cannot dominate.
Qurashi et al (2021) [29]	Overall, most of the participants (83%) were familiar with the machine learning function and AI concept. 82% of participants had never used AI in their departments. 95.5% showed strong interest in AI education and are willing to incorporate it into the clinical practice of radiology.	92.9% of the participants showed willingness towards introducing AI in clinical practice of radiology.	Almost half of radiography students (47.8%) believe that their job might be at risk due to AI application.
Coppola et al (2021) [30]	NR	Radiologists had a mostly positive attitude toward the implementation of AI in their working practice. Overall, 77% of respondents were favorable to the adoption of AI. 73% perceived AI advantages of lowering diagnostic error rate.	Most radiologists (88.9%) were not afraid of losing their job due to it.
Bisdas et al (2021) [31]	Most students (above 56%) claimed at least a moderate understanding of AI. Most respondents (74.6%) were at least aware that AI is being broadly discussed in the medical community. 85.6% agreed that AI shall be part of the medical training.	The belief that AI will revolutionize medicine and dentistry (83.9%) with greater agreement for students from a developed country was noted. Most students agree that the AI developments will make medicine and dentistry more exciting (69.9%) and they are eager to incorporate AI in their future practice (99%).	Students perceived AI as partner rather than as competitor (72.2%). Only the slight majority of the students (56.8%) agreed that AI will never make the human physician expendable. Furthermore, it was common belief in dental students and from respondents in developing countries that non-interventional physician will be replaced.
Tran et al (2021) [32]	Undergraduate medical students in Vietnam had great confidence in the knowledge of their work characteristics, understanding how AI could assist them to promote diagnosis performance, and desire to use AI when available. However, there were still some gaps between their expectancy and preparation, including awareness of technology characteristics and	Overall, the perceptions of our students about diagnosis-related capacities of AI, effort to use AI, and intention to use AI were positive. Effort expectancy and social influence were positively associated with initial trust, while no association was found between performance expectancy and initial trust. Only social influence was positively related to the behavioral intention.	The majority of our sample somewhat agreed or agreed that AI would replace the position of physicians in healthcare.

	capacities to use such technology. Equipping the medical students with the basics, as well as the correct understanding and attitudes about the application of AI in medicine, are crucial.		
Wood et al (2021) [33]	Almost half (45%) of responding students use some kind of AI applications in general. But only 30% of students and 50% of faculty responded that they were aware of AI topics in medicine. Faculty were more likely to report that they did not have a basic understanding of AI technologies. Most agreed that AI should be part of medical education and training.	Most agreed that Artificial intelligence will revolutionize medical practice.	Most agreed that AI will eventually make some medical specialties expendable and new AI developments make medicine, in general, more exciting; slightly disagreed AI technology developments frighten them.
Prakash et al (2021) [34]	NR	The results show that performance expectancy, effort expectancy, social influence, initial trust, and resistance to change predict intention to use. Further, inertia, perceived threat, and risks (medico-legal and performance) determine resistance to change.	NR
Staartjes et al (2020) [35]	In total, 28.5% reported using ML in their clinical practice, and 31.1% in research.	Adoption rates of ML were relatively evenly distributed, with 25.6% for North America, 30.9% for Europe, 33.3% for Latin America and the Middle East, 44.4% for Asia and Pacific and 100% for Africa with only two responses.	NR
Batumalai et al (2020) [36]	Almost half of respondents (46%) strongly disagree/disagree that the current training and educational tools provided by their departments are sufficient to ensure staff do not lose understanding of general underlying principles of radiotherapy with MPs feeling most strongly about this.	Overall, 69% of respondents felt very probably/probably empowered to drive decisions about implementing automated planning processes. 66% thought automation was very important/important.	27% of respondents believe automation will reduce job satisfaction. 71% of respondents strongly agree/agree that automation will cause a loss of skills.
Polesie et al	While 81.5% of responders were aware of AI as	For the entire group, 72.3% agreed or strongly agreed	Only 6% of the responders agreed or strongly

(2020) [37]	an emerging topic in pathology, only 18.8% had either good or excellent knowledge about AI. Among all responders, 22.3% had used AI as a diagnostic aid in real life within pathology and 11% had used AI within dermatopathology in particular. 84.1% thought that AI should be a part of medical training.	that AI will improve dermatopathology.	agreed that the human pathologist will be replaced by AI in the foreseeable future.
Polesie et al (2020) [38]	While 85.1% of respondents were aware of AI as an emerging topic in dermatology, only 23.8% had either good or excellent knowledge about AI within dermatology. 79.8% thought that AI should be a part of medical training.	For the entire group, 77.3% agreed or strongly agreed that AI will improve dermatology. Men showed more excitement and less fear about the use of AI within dermatology, as well as within medicine in general.	Only 5.5% of the respondents agreed or strongly agreed that the human dermatologist will be replaced by AI in the foreseeable future.
Eltorai et al (2020) [39]	All of the radiologists (100%) reported having heard of AI/ML, but only 7.3% reported having read scientific journal articles on AI/machine learning in the past year.	Overall, both chest radiologists and computer scientists are optimistic about the future of AI in radiology. 31.6% and 61.1% of chest radiologists predicted radiologists' job will be dramatically different in 5 to 10 years, and 10 to 20 years, respectively.	0% radiologist expected radiologists to become obsolete in 5 years; 3.2% predicted radiologist obsolescence in 10 to 20 years.
Petitgand et al (2020) [40]	NR	This in-depth case study identified availability, usability and perceived usefulness to be barriers physician adoption of an AI-based decision support system in emergency care.	NR
Shen et al (2020) [41]	In total, 99.51% of participated dermatologists pay attention (general, passive-active, and active attention) to information pertaining to AI.	The majority of Chinese dermatologists are interested in AI information. Nearly all dermatologists are attentive to information on AI and think the role of AI is in "assisting the daily diagnosis and treatment activities for dermatologists".	95.36% of participated dermatologists thought the role of AI to be in "assisting the daily diagnosis and treatment activities for dermatologists", and only 3.42% of participated dermatologists thought AI would replace dermatologists' daily work.
Petkus et al (2020) [42]	NR	Some specialties welcome clinical decision support system (CDSS) and are benefiting from their input to	NR

		support decisions on a range of clinical tasks.	
Doraiswamy et al (2020) [43]	NR	Only 36% felt that the potential benefits of future AI/ML would outweigh the possible risks in their field.	Only 3.8% of respondents felt it was likely that future technology would make their jobs obsolete
Castagno et al (2020) [44]	64% of respondents reported never coming across applications of AI in their work and 87% did not know the difference between machine learning and deep learning, although 50% knew at least one of the two terms. Furthermore, only 5% stated using speech recognition or transcription applications on a daily basis, while 63% never utilize them.	79% believed AI could be useful or extremely useful in their field of work.	Only 10% were worried AI would replace them at their job.
Abdullah et al (2020) [45]	74% agreed with the statement “I have good knowledge of AI.”	74% had high hopes about AI applications in the health care sector, 75% agreed that AI abilities are superior to human experience, and 70% believed AI can speed up the process in health care.	78% were worried AI would replace them at their job.
Blease et al (2020) [46]	NR	Participants were optimistic that technology might improve efficiencies and access to care, and reduce costs. Overwhelmingly, psychiatrists were skeptical that technology could replace human empathy.	Many predicted that ‘man and machine’ would increasingly collaborate in undertaking clinical decisions, with mixed opinions about the benefits and harms of such an arrangement. Participants were optimistic that technology might improve efficiencies and access to care, and reduce costs. But psychiatrists expressed divergent views about the value and impact of future technology with worrying omissions about practice guidelines, and ethical and regulatory issues.
Wadhwa et al (2020) [47]	Of the participants, 85.5 % reported interest in new technologies to assist in colonic polyp detection.	84.7% agreed that computer-assisted polyp detection (CADe) would improve their endoscopic performance.	NR

Sit et al (2020) [48]	44.6% of respondents strongly agreed or agreed that they had an understanding of the basic computational principles that underpin AI. 89% believed that teaching in AI would be beneficial for their careers and 78% agreed that students should receive training in AI as part of their medical degree.	The majority (88%) of respondents believe that AI will play an important role in healthcare in the future, but only 10.4% of students agreed that they would be confident in using AI tools if required.	48.3% of students also believed that some specialties will be replaced by AI within their lifetime. Just under half of the respondents (49.2%) reported they were less likely to consider a career in radiology due to AI.
Bin Dahmash et al (2020) [49]	Approximately 50% believed they had a good understanding of AI; however, when knowledge of AI was tested using five questions, on average, only 22% of the questions were answered correctly. Among the respondents who ranked radiology as their first choice, 58.8% were anxious about the uncertain impact of AI on radiology.	NR	Only 31% believed that AI would replace radiologists in their lifetime, while 44.8% believed that AI would minimize the number of radiologists needed in the future. The number of respondents who ranked radiology as one of their top three choices increased by 14 when AI was not a consideration.
Brandes et al (2020) [50]	64.3% claimed not to have proper knowledge about these new technologies, and 31.7% said they would like more information on the technologies' operation and progress before making a decision on whether or not to practice radiology as a specialty.	A significant proportion of the surveyed students perceive AI as a threat to the radiological practice.	More than half of them (52.5%) said they believe AI poses a threat to the radiology job market. 74.3% reported that they have never considered choosing radiology.
Kasetti et al (2020) [51]	NR	83% of students believed that AI would play a role in medicine. However, a significant number were unsure (14%), and a small proportion (3%) believed AI would play no role in medicine.	Most medical students (54%) believed AI would not be a factor in choosing their subspecialty. Whereas, 25% believed it is a factor and 21% remained unsure. 20% of medical students believed that there would be less doctors in hospital and 15% were unsure.
Sarwar et al (2019) [52]	NR	Overall, respondents carried generally positive attitudes towards AI, with nearly 75% reporting interest or excitement in AI as a diagnostic tool to	Overall, concerns about displacement and negative career impacts were limited; many felt AI would not impact employability (38%)

		facilitate improvements in workflow efficiency and quality assurance in pathology.	or would create new positions and increase employment prospects (42.4%). 19.7% reported being concerned or extremely concerned that AI-tools would displace human jobs. Most respondents felt that diagnostic decision making should remain a predominantly human task (48.3%), or shared equally with an AI algorithm (25.3%), while 20.3% felt that AI-tools should take a dominant role.
Waymel et al (2019) [53]	68.5% did not use any AI software in their daily practice nor foresee any change in the following year. 73.3% estimated they had received insufficient previous information on AI, and 13.7% declared having received no previous specific information at all. Based on the aforementioned criteria, we estimated that 23% had basic knowledge on AI in radiology. 94.4% indicated that they would be interested in receiving a generic training on AI and 69.3% would be interested in receiving a technically advanced training on this subject.	79.3% thought that AI will have a positive impact on their future practice. Respondents agreed upon the fact that AI will revolutionize radiology, affect every field in medicine and improve their daily work.	Respondents disagreed that AI will replace radiologists and had mixed opinions towards the fact that AI was going to shift their activity from diagnostic to interventional radiology.
Gong et al (2019) [54]	78.9% agreed with the statement "I have a good understanding of what AI is", reflecting a high level of confidence in their understanding of AI. However, when developing five statements regarding AI for respondents to answer, 30.7% failed to answer any question correctly and only 14% answered all five questions correctly.	An overwhelming majority (90.2%) of<br>these respondents agreed that "Radiologists should embrace artificial intelligence, and work with the IT industry for its application."	Only a minority (29.3%) of respondents agreed AI would replace radiologists in foreseeable future, but a majority (67.7%) agreed AI would reduce the demand for radiologists. Even among first-choice respondents, 48.6% agreed AI caused anxiety when considering the radiology specialty. Furthermore, one-sixth of respondents who would otherwise rank

			radiology as the first choice would not consider radiology because of the anxiety about AI.
Pinto dos Santos et al (2019) [55]	Around 52% were aware of the ongoing discussion about AI in radiology and 68% stated that they were unaware of the technologies involved. 30.8% thought they had a basic understanding of AI and deep learning as a topic in radiology. Over two-thirds agreed on the need for AI to be included in medical training (71%).	The majority agreed that AI will revolutionise and improve radiology (77% and 86%). In sub-group analyses male and tech-savvy respondents were more confident on the benefits of AI and less fearful of these technologies.	83% disagreed with statements that human radiologists will be replaced. Less than half stated that these developments make radiology or medicine more exciting to them (30.8% and 44.5%, respectively).
Oh et al (2019) [56]	Only 5.9% answered that they had good familiarity with AI.	Most participants considered AI useful in the medical field (83.4% agreement). Less than half of the participants (43.9%) agreed that AI is diagnostically superior to human doctors.	Only 35.4% answered that they agreed that AI could replace them in their jobs.
Blease et al (2019) [57]	NR	GPs' opinions about the potential impact of future technology in primary care were classified into 3 major categories: limitations of future technology, potential benefits of future technology, and social and ethical concerns.	NR
European Society of Radiology (2019) [58]	48% do not currently use AI, 20% use AI, and 30% are planning to do it. 68.8% would like to be educated on advantages and limitations of AI applications.	Responders showed a general favourable attitude towards AI.	Responders foresee AI impact on: job opportunities (56%), expecting increase (58%), reduction (42%); reporting workload (75%), expecting reduction (51%), increase (49%). For all responders, involvement in AI-

			projects is welcome, with different roles: supervision (64%), task definition (53%), image labelling (29%).
Pan et al (2019) [59]	NR	We find support to show that perceived usefulness (PU), attitude, and the experience of using mHealth are key factors that influence both clinicians and non-clinician's adoption intention. Meanwhile, subjective norm has a positive effect on only clinicians' behavioural intention (BI). Among all the constructs, the experience of using mHealth has the strongest positive effect on doctors' adoption intention on smart healthcare services, a positive impact on the PU and perceived ease of use, and a negative impact on the PR.	NR
van Hoek et al (2019) [60]	NR	Surgeons advocate the use of teleradiology. 79% confirmed that their interest in future technologies, such as AI. While the majority of participants agreed that AI should be included as a support system in radiology, surgeons were less supportive than radiologists.	15% of students considered radiology as a possible future specialization. Of those that did not intend to specialize in radiology, 26% stated that AI was one of the reasons. Students saw a potential threat of AI as more likely than radiologists did.

Supplementary Table 2: Distribution of responses to statements regarding perspectives toward clinical AI

Statement	Strongly disagree	Disagree	Neutral	Agree	Strongly agree	*p*-value (physicians vs. medical students)*	*p*-value (low- and lower-middle-income countries vs. high- and upper-middle-income countries)*	*p*-value (have not used clinical AI vs. have used clinical AI)*
I am aware of the wide application of clinical AI	56 (7.39)	112 (14.78)	302 (39.84)	180 (23.75)	108 (14.25)	0.861	0.597	<0.001
I have a good knowledge of clinical AI	127 (16.75)	272 (35.88)	258 (34.04)	78 (10.29)	23 (3.03)	0.333	<0.001	<0.001
I am willing to learn the knowledge about clinical AI proactively	5 (0.66)	13 (1.72)	153 (20.18)	445 (58.71)	142 (18.73)	<0.001	0.139	0.098
I would like hospitals or schools to offer clinical AI related training	5 (0.66)	12 (1.58)	150 (19.79)	441 (58.18)	150 (19.79)	<0.001	0.256	0.275
AI will boost medicine	11 (1.45)	18 (2.37)	175 (23.09)	452 (59.63)	102 (13.46)	0.013	0.113	0.826
AI will be used more and more widely in medicine	9 (1.19)	16 (2.11)	144 (19.00)	433 (57.12)	156 (20.58)	0.006	0.022	0.417
I am willing to use clinical AI if needed	7 (0.92)	14 (1.85)	151 (19.92)	455 (60.03)	131 (17.28)	0.010	0.344	0.351
Clinical AI is more accurate than physicians	48 (6.33)	241 (31.79)	355 (46.83)	102 (13.46)	12 (1.58)	0.004	0.989	0.047
Clinical AI is more efficient than physicians	23 (3.03)	92 (12.14)	248 (32.72)	319 (42.08)	76 (10.03)	<0.001	0.002	0.585
Physicians will be replaced by clinical AI in the future	188 (24.8)	330 (43.54)	181 (23.88)	45 (5.94)	14 (1.85)	0.005	0.002	0.635
Physicians who embrace clinical AI will replace those who do not	38 (5.01)	188 (24.80)	306 (40.37)	181 (23.88)	45 (5.94)	<0.001	0.312	0.107
The development of clinical AI makes me more willing to engage in medicine	19 (2.51)	95 (12.53)	328 (43.27)	270 (35.62)	46 (6.07)	<0.001	0.478	0.064
The development of clinical AI makes medicine less attractive to me	71 (9.37)	310 (40.90)	305 (40.24)	57 (7.52)	15 (1.98)	.226	.637	0.219

*Mann-Whitney U test

Supplementary Table 3: Factors related to physician's willingness to use clinical AI

Characteristics	N (%)	Willingness to use clinical AI	
		**OR (95% CI)**	**aOR (95% CI)***
Age	38.73±8.75**	1.03 (1.00-1.07)	1.08 (1.02-1.16)
Gender			
Male	92 (26.74)	1.00	1.00
Female	252 (73.26)	0.89 (0.47-1.64)	0.79 (0.40-1.51)
Education level			
Bachelor's degree or below	188 (54.65)	1.00	1.00
Master's or higher degree	156 (45.35)	1.08 (0.63-1.88)	0.81 (0.40-1.61)
Hospital level			
Primary or secondary hospital	121 (35.17)	1.00	1.00
Tertiary hospital	223 (64.83)	1.83 (1.05-3.18)	2.16 (1.11-4.25)
Title			
Resident physician	93 (27.03)	1.00	1.00
Attending physician	139 (40.41)	1.27 (0.66-2.42)	1.15 (0.51-2.65)
Associate chief physician or Chief physician	112 (32.56)	1.63 (0.80-3.35)	1.07 (0.33-3.38)
Work experience (years)			
≤10	152 (44.19)	1.00	1.00
>10	192 (55.81)	1.06 (0.61-1.82)	0.46 (0.17-1.18)

*aOR: adjusted odds ratio, adjusted for all variables in the table; CI: Confidence Interval

**mean ± SD

Supplementary Table 4: Factors related to medical student's willingness to use clinical AI

Characteristics	N (%)	Willingness to use clinical AI	
		**OR (95% CI)**	**aOR (95% CI)***
Age	23.90±3.56**	0.98 (0.93-1.05)	1.02 (0.94-1.11)
Gender			
Male	134 (32.37)	1.00	1.00
Female	280 (67.63)	1.19 (0.74-1.88)	1.20 (0.74-1.93)
Major			
Non-clinical medicine	159 (38.41)	1.00	1.00
Clinical medicine	255 (61.59)	0.92 (0.58-1.45)	0.86 (0.54-1.36)
Learning stage			
Undergraduate	231 (55.80)	1.00	1.00
Master or doctoral student	183 (44.20)	0.69 (0.45-1.08)	0.57 (0.31-1.04)
Clinical internship experience			
No	178 (43.00)	1.00	1.00
Yes	236 (57.00)	0.93 (0.59-1.45)	1.15 (0.67-1.99)

*aOR: adjusted odds ratio, adjusted for all variables in the table; CI: Confidence Interval

**mean ± SD

Reference

1. Shelmerdine SC, Rosendahl K, Arthurs OJ. Artificial intelligence in paediatric radiology: international survey of health care professionals' opinions. Pediatr Radiol. 2022;52(1):30-41.
2. Buck C, Doctor E, Hennrich J, Jöhnk J, Eymann T. General practitioners' attitudes toward artificial intelligence–enabled systems: interview study. J Med Internet Res. 2022;24(1):e28916.
3. Abuzaid MM, Elshami W, Tekin H, Issa B. Assessment of the willingness of radiologists and radiographers to accept the integration of artificial intelligence into radiology practice. Acad Radiol. 2022;29(1):87-94.
4. Khafaji MA, Safhi MA, Albadawi RH, Al-Amoudi SO, Shehata SS, Toonsi F. Artificial intelligence in radiology: are Saudi residents ready, prepared, and knowledgeable? Saudi Med J. 2022;43(1):53-60.
5. Lim SS, Phan TD, Law M, Goh GS, Moriarty HK, Lukies MW, et al. Non-radiologist perception of the use of artificial intelligence (AI) in diagnostic medical imaging reports. J Med Imaging Radiat Oncol. 2022.
6. Kansal R, Bawa A, Bansal A, Trehan S, Goyal K, Goyal N, et al. Differences in knowledge and perspectives on the usage of artificial intelligence among doctors and medical students of a developing country: a cross-sectional study. Cureus. 2022;14(1):e21434.
7. Eiroa D, Antolín A, Fernández Del Castillo Ascanio M, Pantoja Ortiz V, Escobar M, Roson N. The current state of knowledge on imaging informatics: a survey among Spanish radiologists. Insights Imaging. 2022;13(1):34.
8. Reeder K, Lee H. Impact of artificial intelligence on US medical students' choice of radiology. Clin Imaging. 2022;81:67-71.
9. Teng M, Singla R, Yau O, Lamoureux D, Gupta A, Hu Z, et al. Health care students' perspectives on artificial intelligence: countrywide survey in canada. JMIR Med Educ. 2022;8(1):e33390.
10. Pangti R, Gupta S, Gupta P, Dixit A, Sati HC, Gupta S. Acceptability of artificial intelligence among Indian dermatologists. Indian J Dermatol Venereol Leprol. 2021;88:232-4.
11. Leenhardt R, Sainz IFU, Rondonotti E, Toth E, Van de Bruaene C, Baltes P, et al. PEACE: Perception and expectations toward artificial intelligence in capsule endoscopy. J Clin Med. 2021;10(23):5708.
12. Hah H, Goldin DS. How clinicians perceive artificial intelligence-assisted technologies in diagnostic decision making: mixed methods approach. J Med Internet Res. 2021;23(12):e33540.
13. Huisman M, Ranschaert E, Parker W, Mastrodicasa D, Koci M, Pinto de Santos D, et al. An international survey on AI in radiology in 1041 radiologists and radiology residents part 2: expectations, hurdles to implementation, and education. Eur Radiol. 2021;31(11):8797-806.
14. Martinho A, Kroesen M, Chorus C. A healthy debate: exploring the views of medical doctors on the ethics of artificial intelligence. Artifi Intell Med. 2021;121:102190.
15. Zheng B, Wu MN, Zhu SJ, Zhou HX, Hao XL, Fei FQ, et al. Attitudes of medical workers in China toward artificial intelligence in ophthalmology: a comparative survey. BMC Health Serv Res. 2021;21(1):1067.
16. Pumplun L, Fecho M, Wahl N, Peters F, Buxmann P. Adoption of machine learning systems for medical diagnostics in clinics: qualitative interview study. J Med Internet Res. 2021;23(10):e29301.
17. Park CJ, Yi PH, Siegel EL. Medical student perspectives on the impact of artificial intelligence on the practice of medicine. Curr Probl Diagn Radiol. 2021;50(5):614-9.
18. Huisman M, Ranschaert E, Parker W, Mastrodicasa D, Koci M, Pinto de Santos D, et al. An international survey on AI in radiology in 1,041 radiologists and radiology residents part 1: fear of replacement, knowledge, and attitude. Eur Radiol. 2021;31(9):7058-66.
19. Zhai H, Yang X, Xue J, Lavender C, Ye T, Li JB, et al. Radiation oncologists' perceptions of adopting an artificial intelligence-assisted contouring technology: model development and questionnaire study. J Med Internet Res. 2021;23(9):e27122.
20. Chen Y, Stavropoulou C, Narasinkan R, Baker A, Scarbrough H. Professionals' responses to the introduction of AI innovations in radiology and their implications for future adoption: a qualitative study. BMC Health Serv Res. 2021;21(1):813.
21. Nelson CA, Pachauri S, Balk R, Miller J, Theunis R, Ko JM, et al. Dermatologists' perspectives on artificial intelligence and augmented intelligence - a cross-sectional survey. JAMA Dermatol. 2021;157(7):871-4.
22. Valikodath NG, Al-Khaled T, Cole E, Ting DSW, Tu EY, Campbell JP, et al. Evaluation of pediatric ophthalmologists' perspectives of artificial intelligence in ophthalmology. J AAPOS. 2021;25(3):164.e1-.e5.
23. Kochhar GS, Carleton NM, Thakkar S. Assessing perspectives on artificial intelligence applications to gastroenterology. Gastrointest Endosc. 2021;93(4):971-5.e2.
24. Scheetz J, Rothschild P, McGuinness M, Hadoux X, Soyer HP, Janda M, et al. A survey of clinicians on the use of artificial intelligence in ophthalmology, dermatology, radiology and radiation oncology. Sci Rep. 2021;11(1):5193.
25. Wong K, Gallant F, Szumacher E. Perceptions of Canadian radiation oncologists, radiation physicists, radiation therapists and radiation trainees about the impact of artificial intelligence in radiation oncology – national survey. J Med Imaging Radiat Sci. 2021;52(1):44-8.
26. Layard Horsfall H, Palmisciano P, Khan DZ, Muirhead W, Koh CH, Stoyanov D, et al. Attitudes of the surgical team toward artificial intelligence in neurosurgery: international 2-Stage cross-sectional survey. World Neurosurg. 2021;146:e724-e30.
27. Cho SI, Han B, Hur K, Mun JH. Perceptions and attitudes of medical students regarding artificial intelligence in dermatology. J Eur Acad Dermatol and Venereol. 2021;35(1):e72-e3.
28. Yurdaisik I, Aksoy SH. Evaluation of knowledge and attitudes of radiology department workers about artificial intelligence. Ann Clin Anal Med. 2021;12(2):186-90.

29. Qurashi AA, Alanazi RK, Alhazmi YM, Almohammadi AS, Alsharif WM, Alshamrani KM. Saudi radiology personnel's perceptions of artificial intelligence implementation: a cross-sectional study. J Multidiscip Healthc. 2021;14:3225-31.
30. Coppola F, Faggioni L, Regge D, Giovagnoni A, Golfieri R, Bibbolino C, et al. Artificial intelligence: radiologists' expectations and opinions gleaned from a nationwide online survey. Radiol Med. 2021;126(1):63-71.
31. Bisdas S, Topriceanu CC, Zakrzewska Z, Irimia AV, Shakallis L, Subhash J, et al. Artificial intelligence in medicine: a multinational multi-center survey on the medical and dental students' perception. Front Public Health. 2021;9:795284.
32. Tran AQ, Nguyen LH, Nguyen HSA, Nguyen CT, Vu LG, Zhang M, et al. Determinants of intention to use artificial intelligence-based diagnosis support system among prospective physicians. Front Public Health. 2021;9:755644.
33. Wood EA, Ange BL, Miller DD. Are we ready to integrate artificial intelligence literacy into medical school curriculum: students and faculty survey. J Med Educ Curric Dev. 2021;8:1-5.
34. Prakash AV, Das S. Medical practitioner's adoption of intelligent clinical diagnostic decision support systems: a mixed-methods study. Inf Manage. 2021;58(7):103524.
35. Staartjes VE, Stumpo V, Kernbach JM, Klukowska AM, Gadjradj PS, Schröder ML, et al. Machine learning in neurosurgery: a global survey. Acta Neurochir (Wien). 2020;162(12):3081-91.
36. Batumalai V, Jameson MG, King O, Walker R, Slater C, Dundas K, et al. Cautiously optimistic: a survey of radiation oncology professionals' perceptions of automation in radiotherapy planning. Tech Innov Patient Support Radiat Oncol. 2020;16:58-64.
37. Polesie S, McKee PH, Gardner JM, Gillstedt M, Siarov J, Neittaanmäki N, et al. Attitudes toward artificial intelligence within dermatopathology: an international online survey. Fron Med. 2020;7:591952.
38. Polesie S, Gillstedt M, Kittler H, Lallas A, Tschandl P, Zalaudek I, et al. Attitudes towards artificial intelligence within dermatology: an international online survey. Br J Dermatol. 2020;183(1):159-61.
39. Eltorai AEM, Bratt AK, Guo HH. Thoracic radiologists' versus computer scientists' perspectives on the future of artificial intelligence in radiology. J Thorac Imaging. 2020;35(4):255-9.
40. Petitgand C, Motulsky A, Denis JL, Régis C. Investigating the barriers to physician adoption of an artificial intelligence- based decision support system in emergency care: an interpretative qualitative study. Stud Health Technol Inform. 2020;270:1001-5.
41. Shen C, Li C, Xu F, Wang Z, Shen X, Gao J, et al. Web-based study on Chinese dermatologists' attitudes towards artificial intelligence. Ann Transl Med. 2020;8(11):698.
42. Petkus H, Hoogewerf J, Wyatt JC. What do senior physicians think about AI and clinical decision support systems: quantitative and qualitative analysis of data from specialty societies. Clin Med (London). 2020;20(3):324-8.
43. Doraiswamy PM, Blease C, Bodner K. Artificial intelligence and the future of psychiatry: Insights from a global physician survey. Artifi Intell Med. 2020;102:101753.
44. Castagno S, Khalifa M. Perceptions of artificial intelligence among healthcare staff: a qualitative survey study. Front Artif Intell. 2020;3:578983.
45. Abdullah R, Fakieh B. Health care employees' perceptions of the use of artificial intelligence applications: survey study. J Med Internet Res. 2020;22(5):e17620.
46. Blease C, Locher C, Leon-Carlyle M, Doraiswamy M. Artificial intelligence and the future of psychiatry: qualitative findings from a global physician survey. Digit Health. 2020;6:1-18.
47. Wadhwa V, Alagappan M, Gonzalez A, Gupta K, Brown JRG, Cohen J, et al. Physician sentiment toward artificial intelligence (AI) in colonoscopic practice: a survey of US gastroenterologists. Endosc Int Open. 2020;8(10):E1379-E84.
48. Sit C, Srinivasan R, Amlani A, Muthuswamy K, Azam A, Monzon L, et al. Attitudes and perceptions of UK medical students towards artificial intelligence and radiology: a multicentre survey. Insights Imaging. 2020;11(1):14.
49. Bin Dahmash A, Alabdulkareem M, Alfutais A, Kamel AM, Alkholaiwi F, Alshehri S, et al. Artificial intelligence in radiology: does it impact medical students preference for radiology as their future career? BJR Open. 2020;2(1):20200037.
50. Brandes GIG, D'Ippolito G, Azzolini AG, Meirelles G. Impact of artificial intelligence on the choice of radiology as a specialty by medical students from the city of São Paulo. Radiol Bras. 2020;53(3):167-70.
51. Kasetti P, Botchu R. The impact of artificial intelligence in radiology: as perceived by medical students. Russ Electron J Radiol. 2020;10(4):179-85.
52. Sarwar S, Dent A, Faust K, Richer M, Djuric U, Van Ommeren R, et al. Physician perspectives on integration of artificial intelligence into diagnostic pathology. NPJ Digit Med. 2019;2(1):28.
53. Waymel Q, Badr S, Demondion X, Cotten A, Jacques T. Impact of the rise of artificial intelligence in radiology: what do radiologists think? Diagn Interv Imaging. 2019;100(6):327-36.
54. Gong B, Nugent JP, Guest W, Parker W, Chang PJ, Khosa F, et al. Influence of artificial intelligence on Canadian medical students' preference for R]radiology specialty: a national survey study. Acad Radiol. 2019;26(4):566-77.
55. Pinto dos Santos D, Giese D, Brodehl S, Chon SH, Staab W, Kleinert R, et al. Medical students' attitude towards artificial intelligence: a multicentre survey. Eur Radiol. 2019;29(4):1640-6.
56. Oh S, Kim JH, Choi SW, Lee HJ, Hong J, Kwon SH. Physician confidence in artificial intelligence: an online mobile survey. J Med Internet Res. 2019;21(3):e12422.
57. Blease C, Kaptchuk TJ, Bernstein MH, Mandl KD, Halamka JD, DesRoches CM. Artificial intelligence and the future of primary care: exploratory qualitative study of UK general practitioners' views. J Med Internet Res. 2019;21(3):e12802.
58. ESR. Impact of artificial intelligence on radiology: a EuroAIM survey among members of the European Society of Radiology. Insights Imaging. 2019;10(1):105.

59. Pan J, Ding S, Wu D, Yang S, Yang J. Exploring behavioural intentions toward smart healthcare services among medical practitioners: a technology transfer perspective. Int J Prod Res. 2019;57(18):5801-20.
60. van Hoek J, Huber A, Leichtle A, Härmä K, Hilt D, von Tengg-Kobligk H, et al. A survey on the future of radiology among radiologists, medical students and surgeons: students and surgeons tend to be more skeptical about artificial intelligence and radiologists may fear that other disciplines take over. Eur J Radiol. 2019;121:108742.
